# Histopathological Spectrum and Yield of Advanced Neoplasia in Opportunistic Colonoscopy Without Organised Screening: A Single-Centre Cross-Sectional Study from Western Romania

**DOI:** 10.3390/diagnostics16203386

**Published:** 2026-10-18

**Authors:** Alina Anton, Dan Brebu, Meda-Ada Bugi, Andreea-Adriana Neamțu, Anca-Maria Căpraru, Nicoleta Zurbău-Anghel, Dana Iovănescu, Teodor-Andrei Maghiar, Paul Pașca, Flaviu-Ionuț Faur, Sorin Dan Chiriac, Dan Iliescu, Bogdan Dan Totolici, Octavian Crețu, Cristina-Adriana Dehelean, Carmen Neamțu

**Affiliations:** 1 Department of Toxicology, “Victor Babeș” University of Medicine and Pharmacy, Eftimie Murgu Square No. 2, 300041 Timișoara, Romania; dolghi.alina@umft.ro (A.A.); cadehelean@umft.ro (C.-A.D.); 2 Research Centre for Pharmaco-Toxicological Evaluation, “Victor Babeș” University of Medicine and Pharmacy, Eftimie Murgu Square No. 2, 300041 Timișoara, Romania; 3 Department X of General Surgery, Faculty of Medicine, “Victor Babeș” University of Medicine and Pharmacy, Eftimie Murgu Square No. 2, 300041 Timișoara, Romania; brebu.dan@umft.ro (D.B.); paul.pasca@umft.ro (P.P.); flaviu.faur@umft.ro (F.-I.F.); 4 Second Clinic of Surgery, “Pius Brînzeu” Clinical County Emergency Hospital Timișoara, Liviu Rebreanu Boulevard No. 156, 300723 Timișoara, Romania; 5 Faculty of Pharmacy, “Vasile Goldiș” Western University of Arad, Liviu Rebreanu Str. No. 86, 310045 Arad, Romania; 6 Department of Pediatrics I, “Louis Țurcanu” Emergency Clinical Hospital for Children, Doctor Iosif Nemoianu Str. No. 2, 300011 Timișoara, Romania; 7 Department of Pathology, “Pius Brînzeu” Clinical County Emergency Hospital Timișoara, Liviu Rebreanu Boulevard No. 156, 300723 Timișoara, Romania; 8 Department of Pathology, Clinical County Emergency Hospital of Arad, Andrenyi Karoly Str. No. 2–4, 310037 Arad, Romania; zurbau.nicoleta@uvvg.ro; 9 Doctoral School, University of Medicine and Pharmacy of Craiova, 200349 Craiova, Romania; mitran.anca@hotmail.com; 10 Department of Pharmacy, Poiana Mare Psychiatry Hospital, Gării Str. No. 40, 207470 Poiana Mare, Romania; 11 Faculty of Medicine, “Vasile Goldiș” Western University of Arad, Liviu Rebreanu Str. No. 86, 310045 Arad, Romania; iovanescu.dana@uvvg.ro (D.I.); totolici.bogdan@uvvg.ro (B.D.T.); 12 Department of Gastroenterology, Clinical County Emergency Hospital of Arad, Andrenyi Karoly Str. No. 2–4, 310037 Arad, Romania; 13 Department of Surgery, Faculty of Medicine and Pharmacy, University of Oradea, Universității Str. No. 1, 410087 Oradea, Romania; teodormaghiar@yahoo.com; 14 Department of Surgery, Pelican Hospital, Corneliu Coposu Str. No. 2, 410450 Oradea, Romania; 15 Discipline of Surgery III, Department X, Faculty of Medicine, “Victor Babeș” University of Medicine and Pharmacy, Eftimie Murgu Square No. 2, 300041 Timișoara, Romania; sorin.chiriac@umft.ro; 16 Department of Surgery I—Clinic of Surgical Semiotics and Thoracic Surgery, Centre for Hepato-Biliary and Pancreatic Surgery, “Victor Babeș” University of Medicine and Pharmacy, Eftimie Murgu Square No. 2, 300041 Timișoara, Romania; dan.iliescu@umft.ro (D.I.); octavian.cretu@umft.ro (O.C.); 17 Doctoral School of Medicine, “Vasile Goldiș” Western University of Arad, Liviu Rebreanu Str. No. 86, 310045 Arad, Romania; neamtu.carmen@uvvg.ro; 18 Department of Surgery I, Clinical County Emergency Hospital of Arad, Andrenyi Karoly Str. No. 2–4, 310037 Arad, Romania; 19 Faculty of Dentistry, “Vasile Goldiș” Western University of Arad, Liviu Rebreanu Str. No. 86, 310045 Arad, Romania

**Keywords:** colorectal cancer, opportunistic colonoscopy, advanced neoplasia, adenoma, histopathology, serrated lesions, tumour grade, selection bias, Romania

## Abstract

**Background/Objectives:** Organised colorectal cancer screening is still being introduced in Romania, and the yield of opportunistic colonoscopy outside it is poorly documented. Our primary aim was to describe the histopathological spectrum and the yield of advanced neoplasia in an opportunistic colonoscopy service; a secondary, exploratory aim was to describe self-reported lifestyle items in a questionnaire subgroup. **Methods:** This study represents a retrospective cross-sectional analysis of consecutive colonoscopies (June 2023–June 2024) at a county hospital outside organised screening, restricted to patients from whom material reached histopathology; the comparison group therefore comprises patients whose sampled lesions were benign, not patients without neoplasia. Advanced neoplasia was defined histologically as adenocarcinoma or adenoma with high-grade dysplasia, without the 10 mm size criterion. A convenience subgroup self-completed a locally developed, unvalidated questionnaire before the procedure; its items were tested against advanced neoplasia and adenocarcinoma with false discovery rate correction. **Results:** Among 274 patients, 45 (16.4%) had adenocarcinoma, 139 (50.7%) adenoma and 72 (26.3%) advanced neoplasia; per examination (*n* = 978), detection was 4.6% for adenocarcinoma and 7.4% for advanced neoplasia. Forty-four of 45 carcinomas were graded as moderately differentiated, and no serrated lesion was separately reported. Age was associated with advanced neoplasia (OR 1.34 per decade, 95% CI 1.01–1.79). Questionnaire data were available for only 79 patients (28.8%), among whom adenocarcinoma was more frequent than among non-respondents (26.6% vs. 12.3%). In this subgroup, obesity was inversely (OR 0.07, 95% CI 0.02–0.28) and reported weight loss positively (OR 6.77, 2.26–20.30) associated with advanced neoplasia, and two coffee items were inversely associated; processed meat, dairy, aspirin and supplement use were not associated after correction. **Conclusions:** Opportunistic colonoscopy at this centre recovered a high proportion of carcinoma, consistent with symptom-driven presentation, and the reporting of serrated lesions and tumour grade requires standardisation. The questionnaire findings rest on a small, selected subgroup and are hypothesis-generating only. The inverse obesity association is most plausibly explained by selection of the comparison group and of respondents, and none of the associations identifies a risk factor.

## 1. Introduction

Colorectal cancer is among the most frequently diagnosed malignancies worldwide and a leading contributor to cancer mortality [1]. Within Europe, mortality has been falling at all ages, by an estimated 4.8% in men and 9.5% in women between 2018 and 2024, although rises have been noted in younger adults in several countries [2]. That aggregate improvement conceals wide variation across member states in incidence, mortality and stage distribution [3], and Romania sits at the unfavourable end of that distribution, with avoidable colorectal cancer mortality substantially above the European Union average [4].

The natural history of the disease is unusually favourable to prevention. Most colorectal carcinomas arise from identifiable precursor lesions through the progressive accumulation of genetic alterations described in the classical adenoma–carcinoma model [5,6], so that detection and removal of precursors at colonoscopy interrupts the sequence before invasion occurs. A second, distinct route—the serrated neoplasia pathway, driven by BRAF or KRAS mutation and the CpG island methylator phenotype—accounts for an estimated 15% to 30% of colorectal cancers and proceeds through sessile serrated lesions rather than conventional adenomas [7]. Both pathways are recognised in the current World Health Organization classification of digestive system tumours [8].

Translating this understanding into organised practice has been slower in Romania than in Western Europe. The 2022 Council Recommendation on cancer screening identifies quantitative faecal immunochemical testing as the preferred modality for referring individuals aged 50 to 74 to colonoscopy [9]. Romania launched an organised, population-based programme (ROCCAS) in four pilot regions with European Commission support, reporting faecal test acceptance of 89% to 99%, adequate bowel preparation in 84% of examinations and a caecal intubation rate of 93%, with expansion towards national coverage planned from 2024 [10]. The clinical benefit is measurable: among Romanian patients with colorectal cancer, diagnosis through the organised programme carried 2.76-fold higher odds of stage I disease and 6.34-fold higher odds of T1 tumours than symptom-driven opportunistic diagnosis [11]. The same gradient is seen across Europe, where stage I disease accounts for 35.7% to 52.7% of screen-detected cancers but only 13.2% to 24.9% of those detected outside screening [12]. In a regional Romanian referral cohort, 77.1% of colorectal cancers were diagnosed at stage III or IV, and the proportion presenting at advanced stage rose rather than fell across the period studied [13].

The centre studied here was not participating in ROCCAS or any other organised programme during the study period. Colonoscopy was therefore *opportunistic*: examinations were driven by symptoms, incidental findings or individual physician referral rather than by systematic invitation of an asymptomatic population. Such practice is not without yield—a Romanian series of asymptomatic patients undergoing opportunistic colonoscopy found adenomas in 39% and carcinoma in 5.4% [14]—but it depends on who presents rather than on who is invited. Even where organised colonoscopy screening is offered, participation and procedural quality are rate-limiting: in a pragmatic randomised trial, only 42.0% of those invited underwent the examination, and the intention-to-screen reduction in 10-year colorectal cancer risk was 18% (risk ratio 0.82, 95% CI 0.70–0.93) [15]. What an opportunistic service actually recovers is therefore informative in its own right, reflecting the referral threshold and the stage at which patients present, and providing a baseline against which any subsequent screening rollout at the same centre can be measured.

Alongside these structural determinants, a substantial body of evidence links colorectal neoplasia to modifiable exposures. Processed meat is a convincing cause of colorectal cancer and greater body fatness a convincing cause, with red meat a probable cause [16]; the International Agency for Research on Cancer classifies processed meat as carcinogenic to humans and red meat as probably carcinogenic, principally on colorectal evidence [17]. A recent diet-wide analysis of 542,778 women identified dietary calcium as the strongest inverse association (relative risk 0.83 per 300 mg/day) and alcohol as the strongest positive one (relative risk 1.15 per 20 g/day) [18], while an umbrella review has argued that the association between obesity and colorectal cancer is systematically underestimated because prediagnostic weight loss is rarely accounted for [19]. Structured collection of such exposures alongside histopathology is uncommon in Romanian centres and was attempted here in a subset of patients, as a secondary and exploratory component of the study.

Our primary objective was to describe the histopathological spectrum recovered by an opportunistic colonoscopy service, including the burden of advanced neoplasia and its detection per examination, in a population in which early-onset disease is an increasing concern internationally [20] and to examine its association with age and sex, the variables available for the whole cohort. A secondary, exploratory objective was to describe self-reported body composition, recent weight change and reported intake in the subgroup of patients who completed a pre-procedure questionnaire, and to examine their association with histologically defined advanced neoplasia, with the explicit understanding that a convenience subgroup of this kind can generate hypotheses but cannot establish risk factors.

## 2. Materials and Methods

### 2.1. Study Design and Setting

This was a retrospective, single-centre, cross-sectional analysis of consecutive colonoscopies performed in the Department of Gastroenterology of the Clinical County Emergency Hospital of Arad, a tertiary county emergency hospital serving Arad County, western Romania. Procedures were identified from the hospital procedure registry using the national codes for flexible colonoscopy to the caecum (32090-00) and to the hepatic flexure (32084-00) and their combinations with biopsy (32090-01, 32084-01) and polypectomy (32093-00, 32087-00). Examinations performed between 10 June 2023 and 14 June 2024 were eligible. The unit was not participating in an organised colorectal cancer screening programme during this period.

### 2.2. Participants

Patients were eligible if colonoscopic material—biopsy specimens or resected polyps—had been submitted for histopathological examination and a report was available. Each patient contributed one record. One consequence of this eligibility rule should be stated at the outset because it conditions every between-group comparison in this paper: material was submitted from 371 of the 978 colonoscopies performed, so the comparison group consists of patients in whom a lesion was seen and sampled and proved benign—hyperplastic or serrated lesions and low-grade adenomas—and not of patients without colorectal neoplasia. Wherever this article contrasts patients with and without advanced neoplasia, the latter group should be read in that restricted sense.

**Figure 1:**
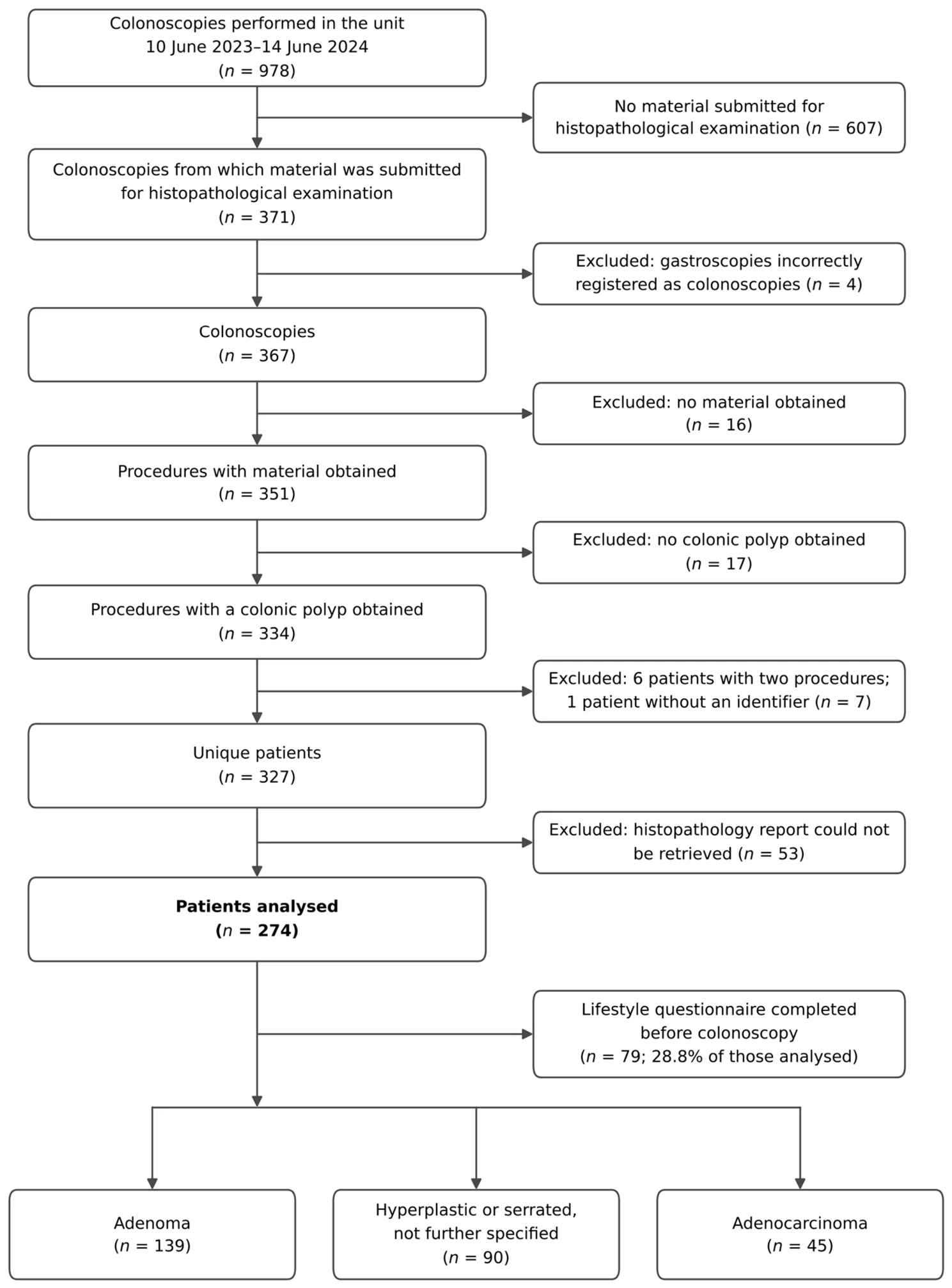
Participant flow, reported in accordance with the STROBE statement. The 978 procedures at the head of the diagram are the complete, unfiltered procedure register for the period: they include examinations of any indication and at any age, examinations that did not reach the caecum, and the four procedures subsequently found to have been gastroscopies miscoded as colonoscopies. Percentages elsewhere in this article use the 274 analysed patients as denominator, except the detection rates per examination in Section 3.2, which use the 978 recorded procedures.

Nine hundred and seventy-eight colonoscopies were recorded in the unit during the study period. This figure is the complete procedure register for the period and has not been filtered in any way: it includes examinations of any indication and at any age, examinations that did not reach the caecum, and the four procedures later found to have been gastroscopies miscoded as colonoscopies. Material was sent for histopathology from 371 of them. Of these, 4 were the miscoded gastroscopies, 16 yielded no material and 17 yielded no colonic polyp, leaving 334 procedures in 327 unique patients. Histopathology reports could not be retrieved for 53 of these patients, who were therefore excluded, giving a final analysed cohort of 274 (Figure 1). The registry total of 978 examinations is the denominator for the detection rates per examination reported in Section 3.2; all other proportions use the 274 analysed patients.

### 2.3. Variables and Definitions

Age and sex were derived from the national personal identification number and cross-checked against the recorded values. The primary outcome was a histopathological diagnosis of adenocarcinoma. Patients were classified by the most advanced lesion recovered into three mutually exclusive categories: hyperplastic or serrated lesion not further specified, adenoma, or adenocarcinoma, following the World Health Organization classification of digestive system tumours [8,21]. Adenomas were characterised by architecture and grade of dysplasia, and carcinomas were characterised by differentiation grade and by descriptive features. A secondary composite outcome, advanced neoplasia, comprised adenocarcinoma together with any adenoma showing high-grade or focally high-grade dysplasia.

Two definitional constraints require explicit statement. First, lesion size was not recorded and could not be retrieved, so the conventional size criterion for advanced neoplasia—an adenoma of 10 mm or more—could not be applied [22]; the composite used here is correspondingly narrower than the term normally implies, and we refer to it throughout as histologically defined advanced neoplasia. Second, sessile serrated lesions and traditional serrated adenomas were not separately distinguished by the reporting pathologists during the study period, and any such lesions would sit within the hyperplastic category, which is therefore labelled throughout as “hyperplastic or serrated, not further specified”.

One reclassification rule was applied before analysis. Two lesions had been reported as intramucosal carcinoma at diagnosis, and a third, originally classified as adenocarcinoma, recorded intramucosal disease among its descriptive features. Because intramucosal colorectal carcinoma is exceptionally uncommon when strict criteria are applied, and because many pathologists prefer the term high-grade dysplasia for such lesions in order to avoid prompting unnecessary surgery without any change in management [23,24], all three were reclassified as adenoma with high-grade dysplasia. They therefore contribute to the adenoma group and to the advanced neoplasia composite, but not to the adenocarcinoma count.

A third variable requires caution. The field recording lesion number was completed from the pathology report and counts specimens submitted rather than lesions seen at endoscopy. Because a carcinoma is typically submitted as a single specimen while entirely benign findings have each polyp enumerated, this variable does not measure the same quantity across histological groups. It is reported descriptively as “specimens submitted” and was deliberately excluded from all modelling.

### 2.4. Lifestyle Questionnaire

A structured questionnaire, headed “semi-quantitative study of dietary habits in colorectal carcinogenesis”, was self-completed by patients before the colonoscopy and before any histopathological result was known. It was written locally for this study, was not adapted from any existing instrument, and was not piloted or validated against any reference measure. It recorded sex, age, height and weight; recent weight loss; coffee, tea, milk and dairy, meat and processed meat consumption; regular aspirin use and dietary supplements; personal and first-degree family history of colorectal neoplasia and inflammatory bowel disease; and the mode and stated purpose of the patient’s presentation. Body mass index was computed from self-reported height and weight. The exact wording of the weight-loss item was “in the recent period, have you noticed a significant decrease in weight?”, answered yes or no, with an optional follow-up recording the number of kilograms lost over the preceding two weeks, one month or three months. Neither “recent period” nor “significant” was defined for the respondent, and the magnitude and window were recorded only when the patient volunteered them, so the variable analysed here is the binary response alone and should be read as a patient’s perception of recent weight change rather than as a measured loss. The remaining consumption items are dichotomous with coarse ordinal follow-ups: coffee and tea in cups per day (one, two to three, more than three), milk and dairy in litres per week (one to two, two to five, more than five), and meat and processed meat as weekly frequency (once, three to four days, daily). The processed-meat item asked specifically about products prepared outside the household. No portion sizes, quantities or energy intakes were captured, and the recall frame was conveyed to respondents only as regular or usual consumption. For that reason, we refer throughout to reported intake rather than to diet or dietary pattern, and no item in this questionnaire should be read as a validated measure of diet in the sense used in nutritional epidemiology. The questionnaire also recorded the mode of presentation (emergency or scheduled) and its stated purpose (screening, symptoms requiring endoscopy, or surgical intervention); these fields are the source of the indication variable reported in Section 3.8, which is consequently available only for respondents and cannot be used to model who completed the questionnaire. The subgroup is a convenience sample in the strict sense. The questionnaire was offered by a single gastroenterologist to patients on that physician’s own lists and only when time allowed; patients attending any other endoscopist during the same period were never offered it. Completion was correspondingly uneven across the study period, with 34 of the 79 questionnaires being completed in the first four months, and the respondents cannot be regarded as a random or representative sample of the cohort.

Exposures were tested against both outcomes. Advanced neoplasia is the better-powered of the two within the subgroup (27 events against 21) and is reported as the main comparison within these analyses; the adenocarcinoma comparison is retained alongside it so that the two can be read together. All questionnaire analyses are secondary and exploratory. They rest on 79 of the 274 patients (28.8%), a convenience sample whose outcome distribution differed from that of non-respondents (Section 3.4), and they are reported to generate hypotheses, not to estimate associations applicable to the cohort or to the wider population.

### 2.5. Statistical Analysis

Continuous variables are summarised as means ± standard deviations and as medians with interquartile ranges and compared using the Mann–Whitney U test. Categorical variables are presented as counts and percentages and compared using the chi-square test without continuity correction, or the Fisher exact test where any observed cell count was below five. Odds ratios are reported with 95% confidence intervals derived from the Woolf standard error, with the Haldane–Anscombe correction applied to zero cells.

Multivariable logistic regression was fitted on the complete cohort with adenocarcinoma and, separately, advanced neoplasia as the dependent variable, and age and sex as covariates. Only variables complete for all 274 patients and measuring the same quantity across groups were entered; the specimen-count variable was excluded for the reason given in Section 2.3.

A second model was fitted within the questionnaire subgroup, with advanced neoplasia as the outcome and body mass index, reported weight loss and age and sex as covariates, restricted to the 62 respondents with complete anthropometry. This age- and sex-adjusted model is the primary subgroup model throughout; its discrimination was summarised by the area under the receiver operating characteristic curve (AUC), with optimism estimated from 2000 bootstrap resamples. Body mass index and reported weight loss were chosen because they showed the strongest unadjusted associations; this is data-driven selection and inflates the resulting estimates, so the estimates should be read with that in mind. Age and sex were entered on prior grounds rather than on the strength of their association in these data. Four covariates were entered against 24 events, which is below the conventional ten events per variable and a further reason to treat the coefficients as descriptive of this subgroup. Ordinal intake variables were additionally tested for dose–response using Spearman rank correlation against the outcome.

Three further analyses were specified after the primary subgroup model had been fitted and are therefore post hoc. First, the body mass index association was re-estimated separately among respondents who did and did not report recent weight loss, both alone and with adjustment for age and sex, to test directly whether reported weight loss accounts for it. Second, the primary subgroup model was refitted without age and sex and, separately, with inverse-probability weights for questionnaire completion, the completion propensity being estimated from age and sex in the full cohort and the weights stabilised; robust standard errors were used for the weighted fit. Third, E-values were computed for the associations surviving correction. Odds ratios were converted to the risk-ratio scale with the square-root approximation because the outcome was not rare in the subgroup. An E-value quantifies the minimum strength of association, on the risk-ratio scale, that an unmeasured confounder would need to have with both the exposure and the outcome, conditional on the measured covariates, to reduce the observed estimate to the null; we report them because the covariates available here are few, and a reader is entitled to ask how much unmeasured confounding would be required before the estimates became uninformative. E-values address confounding only. They say nothing about selection bias, which in this subgroup is the more serious threat, and they should not be read as evidence against it.

For each exposure with a null result, the minimum odds ratio detectable with 80% power at a two-sided alpha of 0.05 was computed from the observed exposure prevalence, outcome prevalence and sample size, so that null findings could be bounded rather than described only as underpowered.

Because thirteen exposures were tested against each outcome and none had been specified in advance as a single primary hypothesis, the exposure analyses are explicitly exploratory. Two-sided *p* values are reported unadjusted and additionally using the Benjamini–Hochberg procedure to control the false discovery rate at 5% [25]. No imputation was performed; analyses of questionnaire variables are complete-case with the denominator stated for every comparison. Analyses were performed in Python 3.11 (pandas, SciPy, statsmodels).

### 2.6. Ethics

This study was conducted in accordance with the Declaration of Helsinki and approved by the Ethics Committee of the Clinical County Emergency Hospital of Arad, Romania under standing approval no. 3388 of 30 January 2020, which covers the use of clinical and histopathological records from this service for research; the examinations analysed here were performed in 2023 and 2024 under that standing approval. Because this study has both a retrospective and a prospective component, the consent arrangements differ between them and are set out separately. The colonoscopies were clinically indicated procedures performed as part of routine care and were not undertaken for research purposes; consent for the medical act itself and for the investigations performed is recorded on the hospital’s standard informed consent form (Acordul pacientului informat), which every patient signs on admission and which is stated to apply across all departments of the hospital together with the risks of the manoeuvres, medication and interventions specific to each speciality. The same form records the patient’s agreement, under Regulation (EU) 2016/679, to the processing of their personal and special-category health data for the conduct of university educational and research activities, which is the basis on which their records were used here. Consent for the use of the resected and biopsied material in medical studies is recorded separately on the pathology requisition form that accompanies every specimen (Annex 4 to Government Decision no. 451/2004), on which the patient indicates whether the biological products collected from them may be used in medical studies. The lifestyle questionnaire was the one prospective element: it was offered before the colonoscopy, and every respondent gave separate written informed consent to complete it and to have their responses linked to their histopathology result. The analysis dataset was pseudonymised before analysis: names, national identification numbers, telephone numbers and exact procedure dates were removed and record order randomised. The manuscript is reported in accordance with the STROBE statement for observational studies [26].

## 3. Results

### 3.1. Cohort Characteristics

Two hundred and seventy-four patients with a histopathological diagnosis were analysed, from colonoscopies performed between 10 June 2023 and 14 June 2024. Monthly volume ranged from 7 to 32 examinations. One hundred and sixty-two patients (59.1%) were male. Mean age was 66.7 ± 10.0 years (median 68, interquartile range 61–74, range 37–92); 255 (93.1%) were aged 50 years or older and 212 (77.4%) were 60 or older, while only 2 (0.7%) were younger than 40. Throughout, proportions are expressed with the 274 patients from whom material was submitted as the denominator; detection rates per examination, which use the 978 colonoscopies performed in the unit during the study period, are reported separately in Section 3.2.

Data quality in the analysed records was high for the core variables: age, sex, lesion type and specimen count were complete for all 274 patients, no duplicate identifiers were present, and recorded sex and year of birth agreed with the national identification number in every case. Completeness fell sharply for the remaining variables (Section 3.7). Characteristics by histological diagnosis are shown in Table 1.

### 3.2. Histopathological Spectrum

Adenomas were the most frequent finding, in 139 patients (50.7%), followed by hyperplastic or serrated lesions in 90 (32.8%) and adenocarcinoma in 45 (16.4%). Among the 139 adenomas, 100 (71.9%) were tubular, 35 (25.2%) tubulovillous and 1 (0.7%) villous, with architecture not specified in the 3 reclassified lesions. Low-grade dysplasia was present in 112 (80.6%), focally high-grade dysplasia in 19 (13.7%) and high-grade dysplasia in 8 (5.8%), so that 27 adenomas (19.4%) carried high-grade change of some degree. Combined with the 45 carcinomas, 72 patients (26.3%) met the study definition of advanced neoplasia.

All 45 carcinomas were assigned a differentiation grade: 44 (97.8%) were moderately differentiated (G2) and 1 (2.2%) poorly differentiated (G3). No well-differentiated (G1) carcinoma was identified. Both the absence of G1 and the near-absence of G3 were verified against the original reports. Descriptive tumour features were recorded for 38 carcinomas (84.4%). Full histopathological detail is given in Table 2.

Relative to the 978 colonoscopies performed in the unit during the study period, these counts give a detection rate per examination of 4.6% for adenocarcinoma (45/978, 95% CI 3.5–6.1), 7.4% for advanced neoplasia (72/978, 95% CI 5.9–9.2) and 14.2% for adenoma (139/978, 95% CI 12.2–16.5). Because histopathology reports were unavailable for 53 patients from whom material had been submitted, each of these is a lower bound; the corresponding upper bounds, obtained by assigning every unretrieved report to the category in question, are 10.0% for adenocarcinoma and 19.6% for adenoma. These figures are not adenoma detection rates in the sense of the European quality indicator, and we do not compare them with it. The denominator here is every procedure recorded in the unit over the period, regardless of any indication or age, including examinations that did not reach the caecum, whereas the quality indicator is defined on screening, surveillance or symptomatic colonoscopies in patients aged 50 years or over; the numerator counts only patients from whom material reached pathology and cannot capture lesions removed without submission. The figures are reported as a description of what this service recovered, not as a performance measure.

### 3.3. Characteristics of the Whole Cohort Associated with Neoplasia

Patients with adenocarcinoma were on average 2.1 years older than those with benign lesions (68.4 ± 9.6 versus 66.3 ± 10.0 years; Mann–Whitney *p* = 0.214), and patients with advanced neoplasia were 2.7 years older (68.7 ± 9.9 versus 66.0 ± 9.9; *p* = 0.044). Sex was associated with neither outcome: adenocarcinoma occurred in 28 of 162 men (17.3%) and 17 of 112 women (15.2%) (OR 1.17, 95% CI 0.61–2.25; *p* = 0.635), and advanced neoplasia in 25.9% of men and 26.8% of women (OR 0.96, 95% CI 0.55–1.65; *p* = 0.888).

A synchronous lesion of different histology was not associated with adenocarcinoma (14.1% versus 17.6%; OR 0.77, 95% CI 0.38–1.55; *p* = 0.466) but was associated with advanced neoplasia (38.0% versus 20.3%; OR 2.41, 95% CI 1.38–4.19; *p* = 0.002), consistent with the recognised association between multiple synchronous lesions and neoplastic risk [22,27].

In logistic regression restricted to variables complete for the whole cohort, age was independently associated with advanced neoplasia (OR 1.34 per decade, 95% CI 1.01–1.79; *p* = 0.044) but not with adenocarcinoma alone (OR 1.24 per decade, 95% CI 0.89–1.73; *p* = 0.210); sex was associated with neither. Both models explained little of the variation, the expected consequence of having no measure of lesion size, site or morphology (Table 3).

### 3.4. Lifestyle Profile of the Questionnaire Subgroup

The questionnaire was self-completed by 79 of the 274 patients (28.8%) before their colonoscopy; among them, 21 (26.6%) were found to have adenocarcinoma and 27 (34.2%) advanced neoplasia, against 12.3% and 23.1% among the 195 who did not complete it (*p* = 0.004 and *p* = 0.059, respectively). Respondents and non-respondents did not differ in age (mean 67.0 versus 66.6 years; *p* = 0.808) or sex (55.7% versus 60.5% male; *p* = 0.463), so the subgroup over-represents carcinoma without being distinguishable on the demographic variables available for the whole cohort. Their reported habits are summarised in Table 4. Coffee was consumed by 60 (75.9%), most commonly a single cup daily; tea by 33 (42.3%); and milk or dairy products by 65 (82.3%), almost always at one to two litres per week. Meat was eaten by 75 (94.9%), daily by 31 (41.3%), and processed meat by 50 (63.3%), daily by 16 (32.0% of consumers). Regular aspirin use was reported by 11 patients (14.1%) and dietary supplement use by 15 (20.8%).

The subgroup had a high mean body mass index: the mean body mass index was 28.6 ± 6.3 kg/m^2^ (median 29.4, IQR 24.2–33.4), with 29 patients (46.0% of the 63 with anthropometry) obese and only 3 (4.8%) underweight. Twenty-one patients (26.9%) answered yes to the weight-loss item, which did not ask whether the loss was intentional.

### 3.5. Lifestyle Exposures and Colorectal Neoplasia

**Figure 2:**
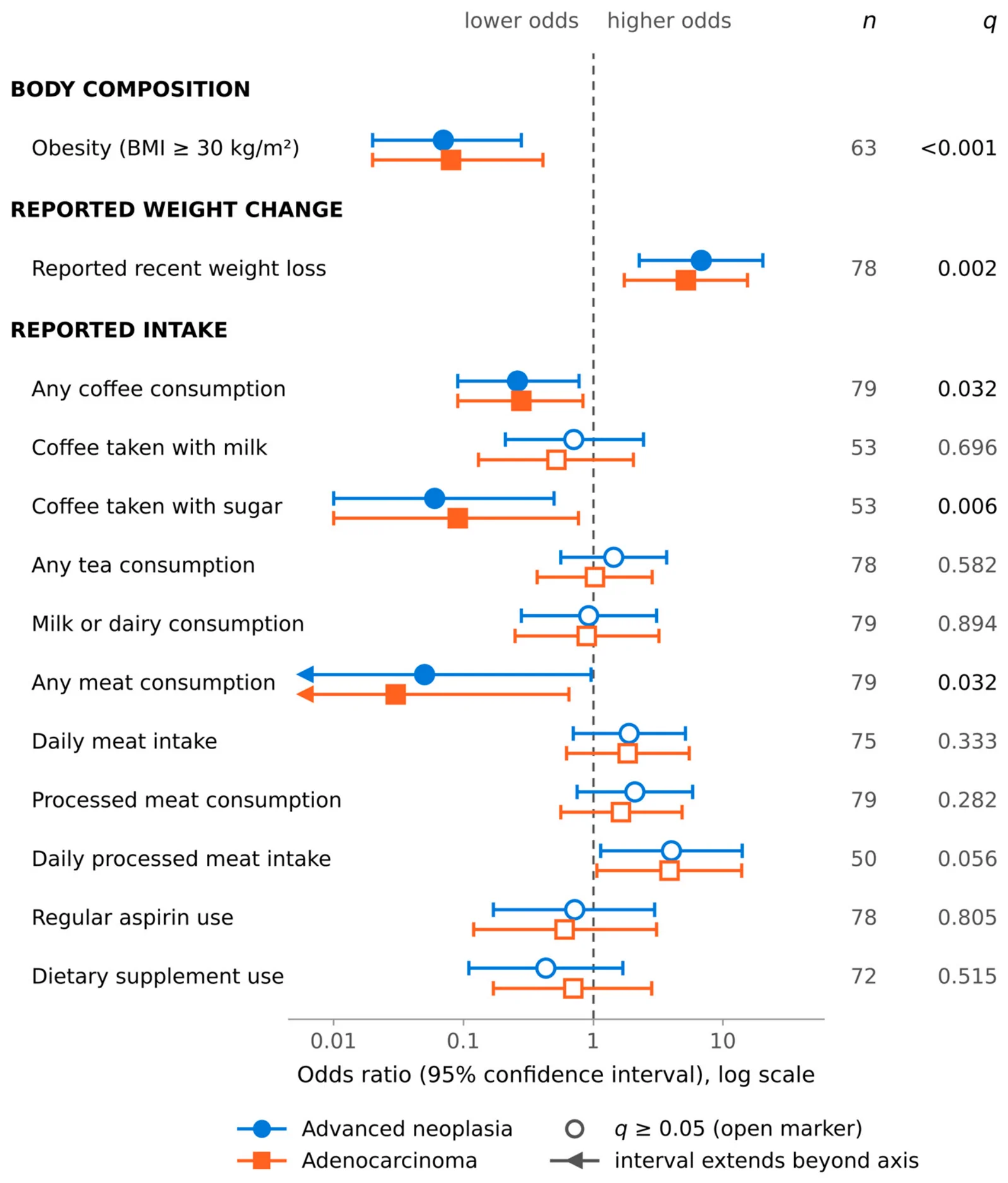
All thirteen self-reported exposures tested against adenocarcinoma and against advanced neoplasia in the questionnaire subgroup, grouped as body composition, reported weight change or reported intake; the grouping is descriptive and implies no interpretation of the individual items. Odds ratios are plotted on a logarithmic scale with 95% confidence intervals; filled markers denote associations surviving Benjamini–Hochberg correction within that outcome, open markers those that do not, and arrowheads intervals extending beyond the axis. *n* is the number of respondents with the exposure recorded; *q* is the false discovery rate-adjusted *p* value against advanced neoplasia. The same estimates are given numerically in Table 5.

Each exposure was tested against both outcomes—adenocarcinoma and the composite of advanced neoplasia—with Benjamini–Hochberg control of the false discovery rate applied separately within each outcome (Figure 2, Table 5). Three exposures were associated with advanced neoplasia at a false discovery rate below 1%: obesity (OR 0.07, 95% CI 0.02–0.28), reported recent weight loss (OR 6.77, 95% CI 2.26–20.30) and taking coffee with sugar (OR 0.06, 95% CI 0.01–0.50). Any coffee consumption (OR 0.26; *q* = 0.032) and meat consumption (OR 0.05; *q* = 0.032) were also associated, the latter resting on only four non-consumers and carrying no information.

Apart from the two coffee items and the uninformative meat item, no item of reported intake was associated with either outcome once corrected. Daily processed meat intake was the only exposure pointing in the direction predicted by the epidemiological literature—present in 62.5% of patients with advanced neoplasia against 29.4% without (OR 4.00, 95% CI 1.14–14.00)—but it did not survive correction (*q* = 0.056) and rests on 50 respondents. Tests for dose–response across the graded intake variables were uniformly null: Spearman correlations with advanced neoplasia were −0.03 for coffee cups per day, +0.04 for tea, +0.15 for weekly milk volume, +0.16 for meat frequency (*p* = 0.18) and +0.22 for processed meat frequency (*p* = 0.13), and the processed-meat gradient was not monotone.

These null results can be bounded. At 80% power and a two-sided alpha of 0.05, and given the exposure prevalences observed, the smallest odds ratio this subgroup could have detected was 4.9 for tea consumption, 5.8 for dietary supplement use and 7.0 for regular aspirin use. For daily processed meat and for dairy consumption, no plausible effect size was detectable at all because 63.3% and 82.3% of respondents, respectively, reported the exposure and the unexposed groups are correspondingly small. The dietary analyses therefore exclude only large effects and are uninformative about associations of the magnitude reported in the prospective literature, which for individual dietary exposures rarely exceed a relative risk of 1.2 per standard increment [18].

Associations were consistently stronger against advanced neoplasia than against adenocarcinoma alone, reflecting the larger number of events (27 versus 21 within the subgroup) rather than any difference in direction.

### 3.6. Multivariable Analysis Within the Questionnaire Subgroup

Because obesity and reported weight loss were the two strongest exposures and are not independent of one another, both were entered into a logistic model with advanced neoplasia as the outcome, together with age and sex, restricted to the 62 respondents with complete anthropometry (24 events). Body mass index and reported weight loss retained independent associations: each 5 kg/m^2^ increase in body mass index was associated with a 75% reduction in the odds of advanced neoplasia (OR 0.25, 95% CI 0.12–0.54; *p* < 0.001), and reported weight loss with a ninefold increase (OR 9.27, 95% CI 1.97–43.66; *p* = 0.005). Neither age (OR 1.61 per decade, *p* = 0.263) nor male sex (OR 0.29, *p* = 0.132) was associated with the outcome. McFadden’s pseudo-R^2^ was 0.419 (Table 6). Because the two exposures were selected on the strength of their unadjusted associations and the model was fitted and evaluated in the same 62 patients, this figure is optimistic and is reported as a description of fit within this subgroup rather than as evidence of generalisable explanatory power.

For this primary model, the apparent AUC was 0.905; bootstrap estimation over 2000 resamples gave a mean optimism of 0.028 and an optimism-corrected AUC of 0.877 (Table 6). We report these figures only as a description of this model in these 62 patients. The two exposures were selected on the strength of their unadjusted associations, the model was fitted and evaluated in the same patients, and the subgroup is a convenience sample from a single endoscopist; optimism correction addresses overfitting, not any of those three problems, and we make no claim of diagnostic performance. Three sensitivity analyses were run on this model (Table 6). Removing age and sex returned body mass index to OR 0.30 per 5 kg/m^2^ (95% CI 0.15–0.59) and reported weight loss to OR 9.94 (2.24–44.15), with pseudo-R^2^ 0.365. Weighting the primary model by the inverse probability of questionnaire completion left the estimates essentially unchanged (body mass index OR 0.24, 95% CI 0.11–0.53; weight loss OR 9.48, 1.64–54.72). That the weights changed nothing is informative but limited: completion was not predicted by age (OR 1.00 per year, *p* = 0.759) or male sex (OR 0.82, *p* = 0.464), so the stabilised weights ranged only from 0.89 to 1.12, and because the primary model already adjusts for both variables, little change was expected. Adding month of procedure to the completion model showed a strong temporal gradient—the odds of completing fell by 15% per month (OR 0.85, 95% CI 0.79–0.92; *p* < 0.001), from 17 of 23 patients in June 2023 to 1 of 12 in December 2023—but weighting on age, sex and month still cannot correct selection that operates through the outcome, and completion was related to the outcome. We state this rather than presenting the weighted estimates as a correction.

**Figure 3:**
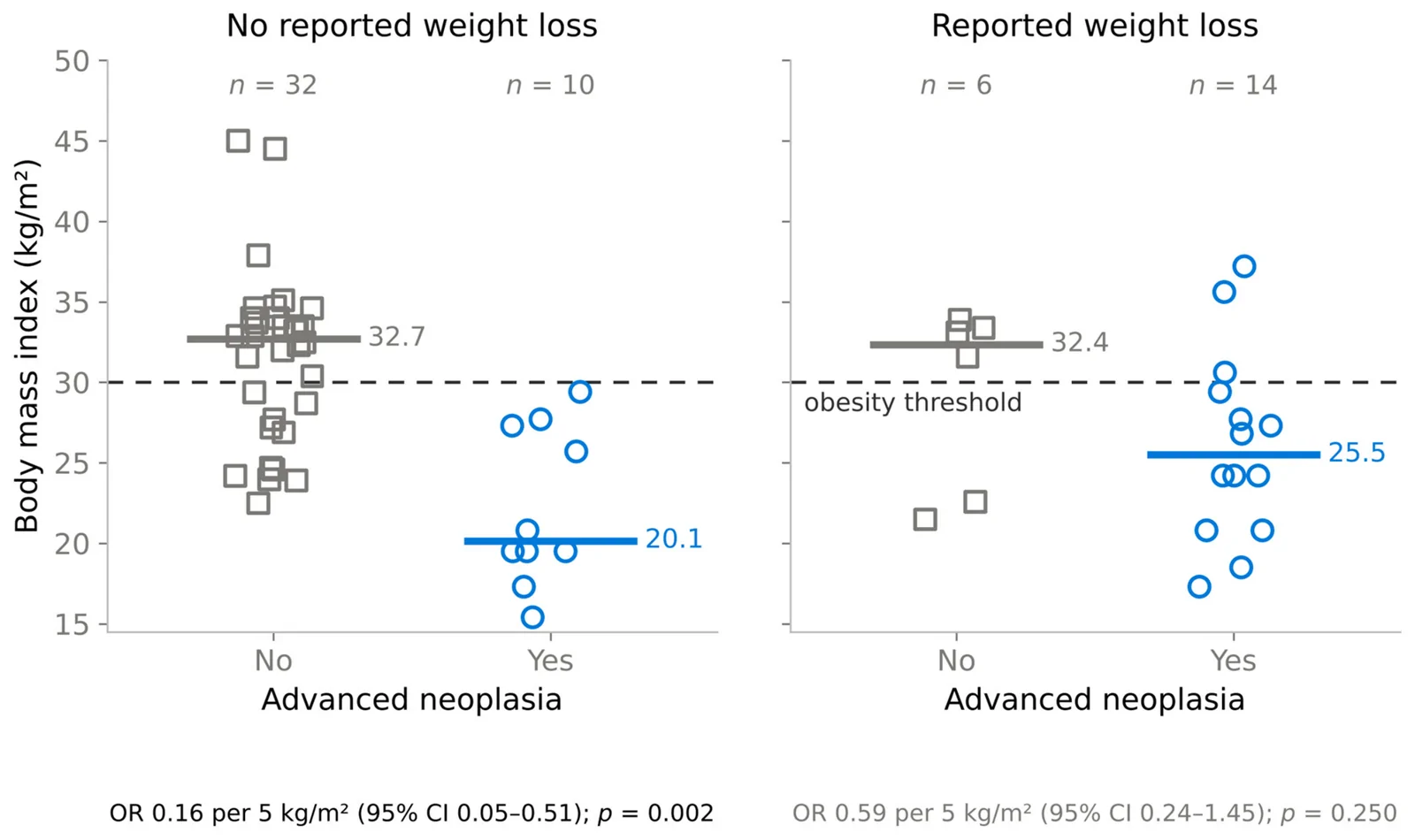
Body mass index by outcome, stratified by reported recent weight loss, among the 62 questionnaire respondents with complete anthropometry. The left panel contains 42 respondents with 10 advanced-neoplasia events and the right panel 20 respondents with 14 events. Each marker is one patient; the horizontal bar is the stratum median, printed beside it; the dashed line is the obesity threshold. Patients with advanced neoplasia are shown as blue circles, the colour and marker used for advanced neoplasia in Figure 2; patients without advanced neoplasia are shown as grey squares. If prediagnostic weight loss were the pathway through which body composition at presentation becomes inverted, the association should be confined to the right-hand panel. It is not: the association is stronger in the panel in which no weight loss was reported. That stratum contains 10 events and no obese patient with advanced neoplasia, so the estimate is unstable; the direction is nonetheless the opposite of what the hypothesised mechanism predicts.

The third analysis was the more consequential. If the inverse body mass index association were produced by prediagnostic weight loss, it should weaken or disappear among respondents who reported no weight loss. It did not. Among the 42 respondents reporting no weight loss, each 5 kg/m^2^ increase in body mass index was associated with an OR of 0.16 for advanced neoplasia (*p* = 0.002; no obese patient in this stratum had advanced neoplasia), whereas among the 20 who did report weight loss, the association attenuated to 0.59 and was not significant (*p* = 0.250); with additional adjustment for age and sex, the estimates were 0.08 (95% CI 0.01–0.43) and 0.55 (0.20–1.53), respectively. The stratum containing the association is the one in which self-reported weight loss is absent, with 10 events and a zero cell, so the estimate itself is unstable; but the direction is unambiguous and it does not support reported weight loss as the mechanism (Figure 3).

E-values were computed for the associations surviving correction. An unmeasured confounder would need to be associated with both exposure and outcome by a risk ratio of at least 7.0 to explain away the obesity association, 4.6 for weight loss and 3.3 for coffee consumption, and by at least 3.2, 2.4 and 1.5, respectively, to move the corresponding confidence limits to the null. Confounding of that strength by a variable unrelated to the disease process is implausible, which points away from residual confounding and towards selection, which E-values do not address.

The direction of the body mass index association is the opposite of that established in prospective epidemiology, where greater body fatness is a convincing cause of colorectal cancer [16,19]. That inversion is the central interpretive problem of this analysis and is addressed in Section 4.6.

### 3.7. Completeness of Recorded Variables

Completeness of every recorded variable in the analysed cohort is given in Table 7.

### 3.8. Indication for Examination

The stated indication was recorded for 75 patients (27.4%) and could not be retrieved for the remainder. Where recorded, 38 examinations (50.7%) were performed for symptoms, 14 (18.7%) were described as colorectal cancer screening, and 23 (30.7%) carried an indication that could not be classified. The proportion with adenocarcinoma did not differ significantly across these groups (11 of 38 symptomatic, 2 of 14 screening, 8 of 23 other; *p* = 0.397), but with fewer than three in ten indications available no conclusion can be drawn.

## 4. Discussion

### 4.1. Principal Findings and Comparison with Romanian Data

In this single-centre series of 274 patients from whom colonoscopic material was submitted for histopathology, adenocarcinoma was found in 16.4% and advanced neoplasia in 26.3%. The cohort was elderly—77.4% were 60 or over and only 6.9% under 50—and predominantly male.

The questionnaire component was secondary and exploratory. It rests on 79 patients (28.8% of the cohort), a convenience sample in which adenocarcinoma was twice as frequent as among non-respondents. Within it, obesity was inversely and reported weight loss positively associated with advanced neoplasia, two coffee items were inversely associated, and none of the exposures with established prior evidence, processed meat included, survived correction for multiple testing. Section 4.6 sets out why we attribute the inverse body mass index association principally to selection of the comparison group and of respondents, and why none of these associations can be read as evidence about risk.

Placed against Romanian comparators, the gradient is instructive. In the South-West Oltenia regional screening programme, invasive adenocarcinoma accounted for 6.2% of lesions examined among 1550 screening colonoscopies [28]; in a Romanian series of asymptomatic patients undergoing opportunistic colonoscopy, carcinoma was found in 5.4% [14]. The proportion here is roughly three times either figure. Two things follow. First, the difference is what would be expected when colonoscopy is driven by symptoms rather than by invitation or by asymptomatic self-referral, and it quantifies for this centre what organised screening is intended to change. Second, the denominators differ: our figure refers to patients from whom a lesion was sampled rather than to all examinations. When referred instead to all 978 colonoscopies performed, adenocarcinoma was detected in 4.6% and advanced neoplasia in 7.4%. Even on that stricter denominator, the carcinoma rate remains close to the 5.4% reported in a Romanian asymptomatic opportunistic series computed on patients rather than examinations [14], and far above what screening colonoscopy yields, so the gradient is not an artefact of how the proportion is expressed.

The distribution of benign lesions is closer to published series. Adenomas represented 60.7% of non-carcinoma lesions here (139 of 229), against 68.8% of analysed polyps in a single-centre series from north-western Romania [29]. Pooled global prevalence estimates give any adenoma in 23.9% and advanced adenoma in 4.6% of individuals examined [30], but those denominators are populations undergoing colonoscopy rather than patients from whom material was recovered, so they are not directly comparable with the figures reported here. A synchronous lesion of different histology was recorded in 28.9% of our carcinoma patients (13 of 45), lower than the 47.4% reported in a comparable single-centre Balkan colonoscopy cohort [31], plausibly because our variable captured only lesions of a different histological type rather than all synchronous polyps.

### 4.2. The Absence of Serrated Lesions

No sessile serrated lesion or traditional serrated adenoma appears anywhere in this dataset; any such lesions are contained within the 90 cases reported as hyperplastic. This is not a peculiarity of one centre. Interobserver agreement between gastrointestinal pathologists in separating sessile serrated lesions from hyperplastic polyps remains only moderate even under the simplified fifth-edition WHO criteria (kappa 0.46–0.63 and 0.44 in two studies) [32,33], reported prevalence varies implausibly between centres—3.0% of 5836 polypectomies in one cohort against 51.4% of lesions in the Oltenia screening programme [28,34]—and the US Multi-Society Task Force grounds part of its surveillance guidance in that variability [35]. Our figure of zero lies at the extreme of that range and is a statement about reporting practice during the study period rather than about the biology of this population. The omission is not trivial: the serrated pathway accounts for an estimated 15% to 30% of colorectal cancers, its lesions are disproportionately proximal and easily missed [7], and it forecloses any examination of the exposures that characterise it. Prospective application of the WHO criteria [8,21] is the single most valuable change to pathology reporting at this centre.

### 4.3. Grading Distribution

The differentiation grades recorded here depart from the expected distribution in both directions. In colorectal adenocarcinoma overall, approximately 70% of tumours are moderately differentiated, with well-differentiated and poorly differentiated tumours accounting for roughly 10% and 20% respectively [23]. In this series, 44 of 45 carcinomas (97.8%) were moderately differentiated, with one poorly differentiated tumour and none well differentiated; both observations were verified against the original reports, so the distribution is not a transcription artefact. At the expected rates, four or five well-differentiated and around nine poorly differentiated tumours would have been anticipated among 45 cases, and the fact that both tails are empty is an observation about grading practice rather than a plausible biological difference. This matters because grade is a stage-independent prognostic variable and because diagnostic variability in this domain is well documented: a national pathology panel found discrepancies in 39.4% of high-grade adenoma cases and 53.0% of early carcinomas [36]. Prospective adoption of the two-tier system, with explicit recording of gland formation, would be a concrete and inexpensive improvement [23].

A related terminological point bears on the cohort definition. Two lesions had been reported as intramucosal carcinoma at diagnosis. The terminology is contested—many pathologists prefer high-grade dysplasia in order to avoid prompting unnecessary surgery, and management is unaffected either way [23]—and a recent re-appraisal applying strict criteria identified only 14 true intramucosal carcinomas in a 34-year archive [24]. A third lesion, classified as adenocarcinoma, recorded intramucosal disease among its descriptive features. The same rule was applied to all three, which are classified as adenomas with high-grade dysplasia (Section 2.3); they contribute to the advanced neoplasia composite but not to the adenocarcinoma count.

### 4.4. Age, Sex and the Adenoma Burden

Age was independently associated with advanced neoplasia but not with adenocarcinoma alone, where the estimate was smaller and the confidence interval crossed unity. With 45 carcinoma events against 72 advanced-neoplasia events, and an age effect of the order of a quarter to a third increase in odds per decade, this study had power to detect the association only for the larger composite. Neither model explained much variation, an expected consequence of having no measure of lesion size, site or morphology.

The absence of any sex difference in carcinoma (17.3% of men versus 15.2% of women) is worth noting, given that men outnumbered women three to two overall and were considerably more likely to have adenomas (64.7% of adenoma patients were male). Male sex is a recognised determinant of adenoma prevalence in pooled analyses [30], and the pattern here suggests the male predominance arose at referral and adenoma detection rather than reflecting differential cancer risk within those referred.

Among adenomas, 19.4% carried high-grade or focally high-grade dysplasia and a quarter were tubulovillous—both higher than would be expected in a screen-detected population, and again consistent with lesions found late. Adenomas were recovered from 14.2% of the 978 examinations performed, rising to at most 19.6% if every unretrieved report is assumed to have described an adenoma. We deliberately do not set this against the 25% adenoma detection rate required by the European Society of Gastrointestinal Endoscopy, because the two quantities are not commensurable: the quality indicator is defined for screening, surveillance or symptomatic colonoscopies in patients aged 50 years or over, whereas our denominator is every procedure recorded in the unit, at any age and for any indication, including examinations that did not reach the caecum, and our numerator counts only lesions that reached pathology. What the figure does establish is a floor for what this service recovered, and the case for recording the adenoma detection rate properly, prospectively and according to the European definition, is strengthened rather than weakened by our inability to compute it here [27,37].

### 4.5. Specimen Count and Lesion Multiplicity

The variable recording lesion number counted specimens submitted to pathology rather than lesions seen at endoscopy, and when entered into a model it produced a strong inverse association between multiplicity and carcinoma. A carcinoma is typically submitted as a single specimen while entirely benign findings have each polyp enumerated, so the variable does not measure the same quantity across groups; we report it descriptively and exclude it from all modelling. No body of evidence supports a protective effect of multiple lesions, whether the endpoint is metachronous advanced neoplasia or cancer incidence [22,38], which is why we treat the finding as an artefact of data capture.

### 4.6. Interpreting the Questionnaire Associations

We regard selection as the most likely explanation for the central questionnaire association, the inverse relationship between body mass index and advanced neoplasia, and it operates at two levels. First, patients without advanced neoplasia are not people free of colorectal disease but patients in whom a lesion was seen, sampled and found benign at a county hospital receiving symptomatic and incidental referrals. Second, the questionnaire reached only patients on one gastroenterologist’s lists, completion fell steeply over the study period, and respondents had twice the carcinoma prevalence of non-respondents (Section 3.4).

The anthropometric data are consistent with selection acting on the comparison group rather than with any property of the patients with neoplasia. Among respondents without advanced neoplasia, 26 of 39 (67%) were obese, with a median body mass index of 32.5 kg/m^2^ (interquartile range 27.4–33.8); among those with advanced neoplasia, 3 of 24 (12%) were obese (median 24.2, interquartile range 19.5–27.7). An obesity prevalence of two-thirds is more than double the 31.9% (95% CI 31.0–32.8) estimated for Romanian adults aged 20 to 79 years in the PREDATORR survey of 2681 participants [39]. That survey covered a wider age range than the present cohort, but the difference is large, and a comparison group enriched for obesity to this extent could on its own produce an inverse association, whatever the relationship in the source population.

The stratified analysis points in the same direction. If the inverse association arose because patients with neoplasia had lost weight before diagnosis, it should be concentrated among respondents who reported weight loss. It was instead stronger among those who reported none (OR 0.16 per 5 kg/m^2^, *p* = 0.002) and attenuated among those who reported weight loss (OR 0.59, *p* = 0.250), and adjustment for age and sex did not change this pattern. The stratum without reported weight loss contains only 10 events and a zero cell, so its estimate is unstable; it is, however, the stratum in which the comparison group was heaviest, with 21 of 32 patients without advanced neoplasia obese, which is what selection of the comparison group would predict. Weighting for questionnaire completion changed none of the estimates, and E-values of 7.0 for obesity and 4.6 for weight loss make ordinary residual confounding an unconvincing alternative; E-values, however, say nothing about selection.

Three further explanations cannot be excluded and may act together with selection. The first is measurement. In prospective data, body mass index measured at diagnosis substantially understates the association between obesity and colorectal cancer, which is clearly positive when body mass index is measured eight to ten years earlier, and prediagnostic weight loss is strongly associated with colorectal cancer close to diagnosis [40]; published obesity estimates are considered systematically underestimated for this reason [19]. A single yes-or-no item asked at the point of colonoscopy, with neither period nor magnitude defined, will miss much of that weight change, and unexpected weight loss has a pooled sensitivity of only 14% as a presenting feature of colorectal cancer [41]. The binary item analysed here is not comparable with the measured weight change in those studies, and we do not compare the estimates numerically. The second is referral: if thinner or frailer patients are referred at a lower threshold, body composition and disease will be associated through the referral decision. The third is chance, in a subgroup of 62 patients with 24 events, with exposures selected on the strength of their unadjusted associations.

The intake associations call for the same reading. Any coffee consumption and taking coffee with sugar were inversely associated with advanced neoplasia, and the meat association rests on four non-consumers. No mechanism links sugar in coffee to protection from colorectal neoplasia, and coffee shows no overall association with colorectal cancer in prospective studies (Section 4.7). The questionnaire did not ask about change in consumption, so these items cannot be interpreted as a reduction in intake caused by illness either. They are most plausibly products of the same selection, of chance among thirteen tested exposures, or of both, and we draw no inference from them.

We therefore report body mass index, reported weight loss and the coffee items as features of this selected subgroup at presentation, not as exposures, and offer no causal reading of any of them. Greater body fatness is a convincing cause of colorectal cancer [16]; a cross-sectional subgroup with a comparison group selected in this way cannot recover that relationship. Distinguishing selection from reverse causation would require a population-based comparison group, weight recorded at referral and again at colonoscopy, and the indication captured for every examination.

As a presenting feature in its own right, unexpected weight loss is highly specific but insensitive, with a pooled specificity of 97% for colorectal cancer in primary care against the sensitivity of 14% noted above [41]. The wider point about alarm symptoms bears directly on how an opportunistic service performs: rectal bleeding carries a pooled positive predictive value of 8.1% among primary-care attenders aged 50 or over [42] but only 0.6% in an unselected general population [43]. Symptom-based case-finding works only in a population already enriched by presentation, which is precisely the population reaching a county hospital endoscopy list, and precisely why it cannot substitute for screening the asymptomatic.

### 4.7. Reported Intake Items with Established Prior Evidence

That none of the intake items with established prior evidence survived correction is not only a matter of power, because the prior evidence for several of these exposures is weaker than is often assumed. A meta-analysis of 26 prospective studies found no overall association for coffee [44], and a pooled analysis of 20 cohorts found none for tea [45]; a network meta-analysis of eleven randomised trials found a reduction with aspirin only at 500 to 1200 mg daily [46], and the US Preventive Services Task Force has removed colorectal cancer prevention from its aspirin recommendation [47]; and a randomised trial of 2259 participants found no effect of calcium or vitamin D supplementation on adenoma recurrence [48]. Processed meat is the exception and the one place where this cohort points where the literature does: daily consumption was reported by 62.5% of patients with advanced neoplasia against 29.4% of those without (OR 4.00, 95% CI 1.14–14.00), the direction that the convincing classification of processed meat as a risk factor would predict [16,17]. It did not survive correction (*q* = 0.056), rests on 50 respondents, and showed no monotone gradient; we report it as the exposure a prospective continuation should be powered to test, not as a finding. Beyond these clinical data, candidate agents continue to be characterised in vitro against colorectal cancer lines, metformin [49] and plant-derived essential oils [50] among them, but none has been tested for neoplasia prevention in a clinical population, and preclinical cytotoxicity is not evidence of a chemopreventive effect.

Several exposures with well-established associations were not captured at all—physical activity [51], alcohol [52] and smoking [53] among them—as were comorbidities such as diabetes that bear on both body habitus and referral. Their absence limits what this questionnaire can say independently of its sample size, and it is not addressed by the E-values, which bound confounding and not selection.

In summary, this study could not have detected a dietary effect of the size the literature reports, and a cross-sectional design measuring reported intake at diagnosis is the wrong instrument for the question at any sample size. The smallest detectable odds ratios were 4.9 for tea, 5.8 for supplements and 7.0 for aspirin, and for daily processed meat and dairy, no plausible effect size was detectable at all because almost every respondent reported the exposure; published associations for individual dietary exposures are an order of magnitude smaller. A null result under these conditions is not evidence of absence. The questionnaire data therefore support no conclusion about diet and colorectal neoplasia, and the associations that were observed are better explained by who presented, who was sampled and who completed the questionnaire than by the exposures themselves.

### 4.8. Clinical Implications

The absence of recorded lesion size prevents this cohort from being risk-stratified according to current practice. The ESGE 2020 guideline makes surveillance decisions turn principally on lesion size (10 mm or more), number (five or more adenomas) and high-grade dysplasia, having explicitly downgraded villous histology as an independent risk factor on the grounds that it does not independently confer increased long-term risk and is subject to high interobserver variability [22]. Only the dysplasia criterion can be applied here, so the appropriate surveillance interval cannot be determined retrospectively for most of these patients. Recording lesion size and colonic segment prospectively is a low-cost change with immediate clinical value.

A second implication concerns the indications that were recorded. Iron deficiency anaemia, a recurring indication in this cohort, is a recognised route to diagnosis: cancer is found in 8% to 10% of adults investigated for it [54], and colorectal cancers diagnosed through an anaemia pathway are more likely to be right-sided (OR 10.61) and more likely to be early stage (OR 1.65) than those presenting symptomatically [55]. With colonic segment unrecorded, this cohort cannot contribute to that question, which is a further argument for capturing site.

The broader implication follows from the yield. A service in which one in six sampled patients has cancer, and in which fewer than one in five recorded indications is screening, is detecting disease rather than preventing it. Romanian data show that diagnosis through the organised programme carries substantially higher odds of stage I and T1 disease than opportunistic diagnosis [11], and the European comparison shows the same gradient across nine countries [12]. Extending organised screening to this county, and repeating this analysis afterwards, would provide a direct before-and-after measure at a single centre. Prevention has a dietary arm as well, and the acceptability of a colorectal cancer-preventive pattern—red meat reduction with increased fibre and micronutrient intake—has been examined separately by this group in Romanian adults [56]. Detection is in any case only the first constraint, since delays between presentation and treatment have been documented for other cancers in the same region [57].

### 4.9. Strengths and Limitations

The strengths are the consecutive, unselected inclusion of patients across a full twelve-month period; the completeness and internal consistency of the core dataset, with age, sex, lesion type and specimen count complete for all 274 patients, no duplicate records and full agreement between recorded demographics and the national identifier; self-completion of the questionnaire before the colonoscopy, which precludes recall influenced by knowledge of the histological result; and the deliberate exclusion from modelling of a variable that does not measure the same quantity across groups.

The limitations are substantial. The design is cross-sectional, so no temporal ordering can be established and no finding should be described as a risk factor. The most important limitation, however, is not the design but the composition of the groups being compared. Material was submitted from 371 of 978 procedures, so patients without advanced neoplasia are not people free of colorectal disease but people in whom a lesion was seen, sampled and found benign; and the questionnaire reached only patients of a single gastroenterologist, with completion falling from 17 of 23 patients in June 2023 to 1 of 12 in December 2023 (odds ratio 0.85 per month, 95% CI 0.79–0.92; *p* < 0.001). Obesity among respondents without advanced neoplasia was twice as prevalent as in the Romanian adult population [39], and we regard this, rather than any property of the patients with neoplasia, as the likeliest source of the inverse body mass index association (Section 4.6). Height and weight were self-reported and show marked digit preference: 36 of 63 recorded weights (57%) are multiples of ten kilograms, and 41 of 63 heights (65%) are multiples of five centimetres, which will misclassify body mass index and, near the threshold, obesity status. Histopathology reports could not be retrieved for 53 of the 327 eligible patients, and selective loss cannot be excluded; the source register from which the cohort was assembled retains no age, sex or usable procedure date for them, which we checked rather than assumed, so the direction of any such loss cannot be assessed. Lesion size, colonic segment, Paris morphology, bowel preparation quality, caecal intubation rate, TNM stage, smoking, alcohol, physical activity and comorbidity including diabetes were not recorded and cannot be retrieved. Serrated lesions were not distinguished during the study period, and the differentiation grades recorded depart from the expected distribution at both tails. The indication was recorded on the questionnaire and so exists only for respondents. The questionnaire subgroup comprised 28.8% of the cohort, was a convenience sample from one endoscopist, used a locally developed and unvalidated instrument, and had a completion rate related to the outcome. Because 71.2% of the cohort had no questionnaire data and missingness was related to the outcome, the subgroup estimates may be biased in either direction and cannot be generalised to the cohort; they are presented as hypothesis-generating only. Histopathology was reported in routine practice without central review, and diagnostic variability in this domain is well documented [32,33,36]. This is a single county hospital, and its referral pattern will not generalise to primary-care or screening populations. Finally, with 45 carcinoma events, this study could reliably detect only large effects, and the numerous non-significant findings should be read as inconclusive rather than as evidence of no association.

## 5. Conclusions

Among 274 patients at a Romanian county hospital from whom colonoscopic material was submitted for histopathology over one year, 16.4% had adenocarcinoma and 26.3% met a histological definition of advanced neoplasia that omits the conventional size criterion and is therefore not directly comparable with the standard definition. Referred to all 978 examinations performed, adenocarcinoma was detected in 4.6% and advanced neoplasia in 7.4%, and adenomas were recovered from between 14.2% and 19.6% of examinations.

The questionnaire component was secondary and exploratory and rests on 28.8% of the cohort, a convenience sample in which carcinoma was over-represented. In that subgroup, obesity was inversely associated with advanced neoplasia, whereas reported weight loss was positively associated with it. Selection is the most likely explanation for the inverse association: the comparison group comprised patients whose sampled lesions proved benign and was obese to an extent that the general population would not predict, and respondents differed from non-respondents in outcome. Our stratified analysis did not support reported weight loss as the pathway, and measurement error in a single self-reported item, referral thresholds and chance remain possible contributors. Reported intake items, processed meat included, provided no interpretable evidence, and the inverse coffee associations have no plausible causal reading. None of these findings identifies a risk factor, and none should be used to inform dietary advice.

Three features of routine practice emerged alongside this: no sessile serrated lesion was separately identified in a full year of specimens, 44 of 45 carcinomas were graded as moderately differentiated, leaving both tails of the grading distribution empty, and adenoma recovery per examination was low. All three are specific and correctable and locate this centre within a documented international problem rather than presenting it as an outlier.

These data describe what opportunistic colonoscopy recovers in the absence of organised screening, and they provide a baseline against which the effect of screening at this centre can later be measured. They do not, and given the cross-sectional design cannot, identify modifiable risk factors for colorectal neoplasia.

## Figures and Tables

**Table 1:** Demographic and endoscopic characteristics by histopathological diagnosis (*n* = 274).

Characteristic	Hyperplastic/Serrated (*n* = 90)	Adenoma (*n* = 139)	Adenocarcinoma (*n* = 45)	Total (*n* = 274)
Age, mean ± SD (years)	64.7 ± 10.5	67.4 ± 9.6	68.4 ± 9.6	66.7 ± 10.0
Age, median (IQR)	67 (56–73)	68 (62–74)	69 (65–74)	68 (61–74)
Male, *n* (%)	44 (48.9)	90 (64.7)	28 (62.2)	162 (59.1)
Female, *n* (%)	46 (51.1)	49 (35.3)	17 (37.8)	112 (40.9)
Age < 50 years, *n* (%)	9 (10.0)	7 (5.0)	3 (6.7)	19 (6.9)
Age 50–69 years, *n* (%)	48 (53.3)	72 (51.8)	22 (48.9)	142 (51.8)
Age ≥ 70 years, *n* (%)	33 (36.7)	60 (43.2)	20 (44.4)	113 (41.2)
Single specimen submitted, *n* (%) †	47 (52.2)	49 (35.3)	34 (75.6)	130 (47.4)
Multiple specimens submitted, *n* (%) †	43 (47.8)	90 (64.7)	11 (24.4)	144 (52.6)
Synchronous lesion of different histology, *n* (%)	5 (5.6)	74 (53.2)	13 (28.9)	92 (33.6)

SD, standard deviation; IQR, interquartile range. † Counts refer to specimens submitted to pathology, not lesions seen at endoscopy; they are not comparable between histological groups and excluded from all modelling (Section 2.3).

**Table 2:** Histopathological characteristics of the lesions (*n* = 274 patients).

Finding	*n*	% of Cohort	% of Group
Principal diagnosis			
Adenoma	139	50.7	—
Hyperplastic/serrated, not further specified	90	32.8	—
Adenocarcinoma	45	16.4	—
Adenoma architecture (*n* = 139)			
Tubular	100	36.5	71.9
Tubulovillous	35	12.8	25.2
Villous	1	0.4	0.7
Not specified	3	1.1	2.2
Adenoma dysplasia grade (*n* = 139)			
Low grade	112	40.9	80.6
Focally high grade	19	6.9	13.7
High grade	8	2.9	5.8
Carcinoma differentiation (*n* = 45)			
Well differentiated (G1)	0	0.0	0.0
Moderately differentiated (G2)	44	16.1	97.8
Poorly differentiated (G3)	1	0.4	2.2
Composite outcome			
Advanced neoplasia	72	26.3	—

Group percentages are calculated within the denominator shown in parentheses. Advanced neoplasia comprises adenocarcinoma and adenoma with high-grade or focally high-grade dysplasia; the conventional size criterion could not be applied (Section 2.3). Three lesions carrying the descriptor intramucosal were reclassified as adenoma with high-grade dysplasia before analysis (Section 2.3); their architecture is recorded as not specified.

**Table 3:** Multivariable logistic regression in the whole cohort (*n* = 274).

Variable	Adjusted OR	95% CI	*p*	Pseudo-R^2^
Outcome: adenocarcinoma (45 events)				0.008
Age (per 10 years)	1.24	0.89–1.73	0.210	
Male sex	1.17	0.61–2.27	0.635	
Outcome: advanced neoplasia (72 events)				0.014
Age (per 10 years)	1.34	1.01–1.79	0.044	
Male sex	0.96	0.55–1.67	0.888	

OR, odds ratio; CI, confidence interval. Models include only variables complete for the full cohort. The specimen-count variable was excluded because it does not measure the same quantity across histological groups (Section 2.3); its inclusion produced a spurious and biologically implausible inverse association with malignancy.

**Table 4:** Self-reported lifestyle profile of the questionnaire subgroup (*n* = 79).

Variable	*n*/Total	%	Notes
Coffee			
Any coffee consumption	60/79	75.9	—
1 cup per day	34/60	56.7	of consumers
2–3 cups per day	23/60	38.3	of consumers
More than 3 cups per day	3/60	5.0	of consumers
Taken with milk	26/53	49.1	where recorded
Taken with sugar	23/53	43.4	where recorded
Tea			
Any tea consumption	33/78	42.3	—
1 cup per day	19/32	59.4	of consumers
Herbal rather than fruit	15/24	62.5	where type recorded
Milk and dairy			
Any consumption	65/79	82.3	—
1–2 L per week	51/64	79.7	of consumers
More than 2 L per week	13/64	20.3	of consumers
Meat			
Any consumption	75/79	94.9	—
Daily	31/75	41.3	of consumers
3–4 days per week	38/75	50.7	of consumers
Processed meat			
Any consumption	50/79	63.3	—
Daily	16/50	32.0	of consumers
3–4 days per week	28/50	56.0	of consumers
Medication and supplements			
Regular aspirin	11/78	14.1	—
Dietary supplements	15/72	20.8	—
Anthropometry and weight change			
Body mass index ≥ 30 kg/m^2^	29/63	46.0	of those measured
Body mass index 25–29.9 kg/m^2^	13/63	20.6	of those measured
Body mass index 18.5–24.9 kg/m^2^	18/63	28.6	of those measured
Body mass index < 18.5 kg/m^2^	3/63	4.8	of those measured
Reported recent weight loss	21/78	26.9	—

Denominators differ because not every item was completed by every respondent; the denominator is stated for each. Body mass index was computed from self-reported height and weight.

**Table 5:** Lifestyle exposures tested against adenocarcinoma and against advanced neoplasia in the questionnaire subgroup.

Exposure	*n*	Carcinoma in Exposed	OR (95% CI)	*p*	*q*	OR (95% CI)	*p*	*q*
Coffee consumption	79	12/60 (20%)	0.28 (0.09–0.83)	0.019	0.048	0.26 (0.09–0.78)	0.012	0.032
Coffee taken with milk	53	4/26 (15%)	0.52 (0.13–2.04)	0.501	0.723	0.71 (0.21–2.44)	0.589	0.696
Coffee taken with sugar	53	1/23 (4%)	0.09 (0.01–0.77)	0.015	0.048	0.06 (0.01–0.50)	0.001	0.006
Tea consumption	78	9/33 (27%)	1.03 (0.37–2.84)	0.952	1.000	1.44 (0.56–3.69)	0.447	0.582
Milk or dairy consumption	79	17/65 (26%)	0.89 (0.25–3.20)	1.000	1.000	0.92 (0.28–3.08)	0.894	0.894
Meat consumption †	79	17/75 (23%)	0.03 (0.00–0.65)	0.004	0.017	0.05 (0.00–0.96)	0.012	0.032
Daily meat intake	75	9/31 (29%)	1.84 (0.62–5.48)	0.269	0.500	1.89 (0.70–5.12)	0.205	0.333
Processed meat consumption	79	15/50 (30%)	1.64 (0.56–4.85)	0.367	0.596	2.10 (0.75–5.82)	0.152	0.282
Daily processed meat intake	50	8/16 (50%)	3.86 (1.07–13.94)	0.034	0.074	4.00 (1.14–14.00)	0.026	0.056
Regular aspirin use	78	2/11 (18%)	0.60 (0.12–3.07)	0.719	0.882	0.72 (0.17–2.97)	0.743	0.805
Dietary supplement use	72	3/15 (20%)	0.70 (0.17–2.83)	0.746	0.882	0.43 (0.11–1.69)	0.356	0.515
Obesity (BMI ≥ 30 kg/m^2^)	63	2/29 (7%)	0.08 (0.02–0.41)	<0.001	0.007	0.07 (0.02–0.28)	<0.001	<0.001
Reported recent weight loss	78	11/21 (52%)	5.17 (1.73–15.46)	0.002	0.014	6.77 (2.26–20.30)	<0.001	0.002

Columns 4–6 give the comparison against adenocarcinoma and columns 7–9 against advanced neoplasia. OR, odds ratio; CI, confidence interval. The *p* values are two-sided and unadjusted; *q* values are Benjamini–Hochberg adjusted across the thirteen exposures, separately within each outcome. *n* is the number of respondents with the exposure recorded. † Only 4 of 79 respondents reported not eating meat, all 4 of whom had adenocarcinoma; the estimate is numerically unstable and should not be interpreted.

**Table 6:** Multivariable logistic regression within the questionnaire subgroup and its sensitivity analyses: advanced neoplasia (*n* = 62, 24 events).

Model	Variable	Adjusted OR	95% CI	*p*
Primary (adjusted for age and sex)	Body mass index (per 5 kg/m^2^)	0.25	0.12–0.54	<0.001
	Reported recent weight loss	9.27	1.97–43.66	0.005
	Age (per decade)	1.61	0.70–3.70	0.263
	Male sex	0.29	0.06–1.45	0.132
	Pseudo-R^2^ 0.419; AUC 0.905 apparent, 0.877 optimism-corrected			
Without age and sex	Body mass index (per 5 kg/m^2^)	0.30	0.15–0.59	<0.001
	Reported recent weight loss	9.94	2.24–44.15	0.003
Primary model weighted for completion	Body mass index (per 5 kg/m^2^)	0.24	0.11–0.53	<0.001
	Reported recent weight loss	9.48	1.64–54.72	0.012
Stratified by weight loss (body mass index only)	Body mass index, no weight loss reported (*n* = 42, 10 events)	0.16	0.05–0.51	0.002
	Body mass index, weight loss reported (*n* = 20, 14 events)	0.59	0.24–1.45	0.250

OR, odds ratio; CI, confidence interval; AUC, area under the receiver operating characteristic curve. All models are restricted to respondents with complete height and weight data. The primary model is adjusted for age and sex and carries 24 events against four covariates (six events per variable), below the conventional threshold of ten; the model without age and sex carries twelve events per variable, and the stratified estimates fewer still. Confidence intervals are therefore wide, and every estimate should be read as descriptive of this subgroup rather than as a generalisable effect size. Pseudo-R^2^ was 0.419 for the primary model and 0.365 for the model without age and sex. The AUC refers to the primary model; optimism was estimated from 2000 bootstrap resamples and corrects for overfitting only, not for selection. Completion weights are stabilised inverse probabilities from a logistic model of questionnaire completion on age and sex in the full cohort; the weighted model was fitted with robust standard errors. Stratified estimates are for body mass index alone; with additional adjustment for age and sex they were 0.08 (95% CI 0.01–0.43) without and 0.55 (0.20–1.53) with reported weight loss. In the stratum reporting no weight loss, no obese patient had advanced neoplasia, so the odds ratio for obesity in that stratum is Haldane-corrected and unstable; the continuous estimate is reported instead.

**Table 7:** Completeness of recorded variables in the analysed cohort (*n* = 274).

Variable	Recorded, *n*	Missing, %	Consequence for the Analysis
Age, sex, lesion type, specimen count	274	0.0	Complete; supports the primary analysis
Date of examination	274	0.0	Complete
Adenoma/carcinoma architecture	181	33.9	Complete within the relevant groups
Dysplasia or differentiation grade	184	32.8	Complete within the relevant groups
Tumour descriptive features	38	86.1	Descriptive only
Height and weight (BMI)	63	77.0	Anthropometric analyses rest on 63 patients
Coffee, dairy, meat, processed meat	79	71.2	Exploratory only
Tea, aspirin, weight loss	78	71.5	Exploratory only
Dietary supplements	72	73.7	Underpowered
Coffee preparation, milk and sugar	53	80.7	Exploratory only
Processed meat frequency	50	81.8	Exploratory only
Indication for examination	75	72.6	Screening and symptomatic referrals cannot be separated
Personal medical history	57	79.2	Comorbidity cannot be adjusted for
Family history of malignancy	55	79.9	Not interpretable

BMI, body mass index. Percentages are of the 274 analysed patients; questionnaire items are missing for the 195 patients who did not complete it.

## Data Availability

The de-identified dataset supporting the reported results is provided in full as Supplementary Table S4b, together with its data dictionary in Table S4a; a machine-readable copy is available from the corresponding author on request. The original clinical records contain direct identifiers and cannot be shared.

## Supplementary Materials

The following supporting information can be downloaded at: https://www.mdpi.com/article/10.3390/diagnostics16203386/s1. Table S1: STROBE checklist for cross-sectional studies; Table S2: Items not reportable from the source data; Table S3: Lifestyle questionnaire as administered; Table S4a: Data dictionary for the analysis dataset; Table S4b: De-identified analysis dataset (*n* = 274).
