# Supplementary material for: Histopathological Spectrum and Yield of Advanced Neoplasia in Opportunistic Colonoscopy Without Organised Screening: A Single-Centre Cross-Sectional Study from Western Romania
Source: Diagnostics (Basel). 2026 Oct 18;16(20):3386. doi: 10.3390/diagnostics16203386 (PMC13703055; doi:10.3390/diagnostics16203386)
Supplement: Supplementary file 1 [file diagnostics-16-03386-s001.zip › diagnostics-4589015-supplementary.pdf]

Supplementary Materials

Histopathological Spectrum and Yield of Advanced Neoplasia in Opportunistic Colonoscopy Without Organised Screening: A Single-Centre Cross-Sectional Study from Western Romania

---

This file contains all supplementary material for the manuscript: the reporting checklist, the questionnaire as administered, the data dictionary, and the complete de-identified analysis dataset. It is self-contained; no other supplementary file is required.

Every estimate reported in the manuscript can be reproduced from the dataset in Table S4b. The cohort comprises 274 patients: 45 with adenocarcinoma, 139 with adenoma and 90 with a hyperplastic or serrated lesion, of whom 72 meet the study definition of advanced neoplasia. Three lesions carrying the descriptor intramucosal are classified as adenoma with high-grade dysplasia throughout, in accordance with the rule stated in Section 2.3.

Table S1. STROBE checklist for cross-sectional studies

Section numbers refer to the manuscript.

Item	Recommendation	How it is addressed	Section
1	Title and abstract	The title names the setting and the design; the abstract states the design in its first Methods sentence and is structured as Background/Objectives, Methods, Results, Conclusions.	Title; Abstract
2	Background / rationale	European and Romanian colorectal cancer burden, the two carcinogenesis pathways, the status of organised screening in Romania, and the rationale for describing an opportunistic cohort.	1
3	Objectives	Primary objective: the histopathological spectrum and the yield of advanced neoplasia recovered by the service, with age and sex examined in the whole cohort. Secondary, exploratory objective: self-reported questionnaire items in the subgroup of respondents, stated in advance to be hypothesis-generating only.	1, final paragraph
4	Study design	Retrospective, single-centre, cross-sectional analysis, stated in the first line of Methods.	2.1
5	Setting	Tertiary county emergency hospital, Arad County, western Romania; procedure codes given; recruitment 10 June 2023 – 14 June 2024; the unit was not in an organised screening programme.	2.1
6	Participants	Eligibility: colonoscopic material submitted for histopathology with a report available. One record per patient. The comparison group therefore comprises patients whose sampled lesions proved benign, not patients without neoplasia. Full exclusion sequence given, with Figure 1 as the flow diagram.	2.2; Figure 1
7	Variables	Two outcomes, adenocarcinoma and histologically defined advanced neoplasia. Thirteen questionnaire exposures, grouped descriptively into body composition, reported weight change and reported intake. The specimen-count variable is defined and its exclusion justified. The reclassification of three intramucosal lesions is stated.	2.3, 2.4
8	Data sources / measurement	Age and sex from the national identifier, cross-checked. Histopathology from routine reports. Questionnaire self-completed before the procedure; provenance, item wording and response options described.	2.3, 2.4

Item	Recommendation	How it is addressed	Section
9	Bias	Selection of the comparison group and of questionnaire respondents named as the leading explanation for the questionnaire associations; respondents compared with non-respondents; completion modelled on age, sex and month. Reverse causation tested by stratifying on reported weight loss, without support for that mechanism. E-values reported with their limits. Unretrievable reports named as possible selective loss.	2.4, 2.5, 3.4, 3.6, 4.6, 4.9
10	Study size	No a priori sample size calculation; the cohort is all eligible patients in a fixed twelve-month period. Minimum detectable odds ratios reported for every null exposure.	2.2, 2.5, 3.5, 4.7, 4.9
11	Quantitative variables	Age continuous and banded; BMI continuous (per 5 kg/m^2) and against the obesity threshold; categorical exposures dichotomised as recorded, with dose–response tested where the response was graded.	2.3, 2.5
12	Statistical methods	Tests named per variable type; odds ratios with Woolf standard errors and Haldane–Anscombe correction; multivariable logistic regression in the whole cohort and, in the questionnaire subgroup, a primary model adjusted for age and sex; Benjamini–Hochberg control within each outcome; three post hoc sensitivity analyses; bootstrap optimism-corrected AUC; E-values; minimum detectable odds ratios; complete-case analysis; software named.	2.5
13	Participants (results)	Numbers at each stage in text and Figure 1: 978 recorded procedures → 371 with material submitted → 367 → 351 → 334 → 327 unique patients → 274 analysed. The 978 is the complete unfiltered register.	2.2, 3.2; Figure 1
14	Descriptive data	Characteristics by histological group (Table 1); lifestyle profile of the subgroup (Table 4), with respondents compared with non-respondents (Section 3.4); completeness of every variable (Table 7).	3.1, 3.4, 3.7
15	Outcome data	Counts for each histological category and for the composite, with denominators (Table 2).	3.2; Table 2
16	Main results	Adjusted estimates from the whole-cohort models (Table 3). Every questionnaire exposure against both outcomes with odds ratios, confidence intervals and unadjusted and adjusted p values (Table 5); the primary age- and sex-adjusted subgroup model with its event count, pseudo-R^2 and apparent and optimism-corrected discrimination (Table 6).	3.3, 3.5, 3.6
17	Other analyses	Questionnaire subgroup reported separately and labelled exploratory; each exposure carries its own denominator; three post hoc sensitivity analyses reported in full, including the stratified analysis.	3.4–3.6, 3.8; Table 6
18	Key results	Summarised with reference to the primary and secondary objectives at the start of the Discussion.	4.1
19	Limitations	A dedicated section naming the composition of the comparison group, single-endoscopist convenience sampling, the 71.2% of the cohort without questionnaire data, digit preference in self-reported anthropometry, the unretrievable reports, uncollected variables, reporting practice, absence of central review, single-centre setting and power.	4.9
20	Interpretation	Selection named as the most likely explanation for the questionnaire associations and set out first; reverse causation, measurement and referral considered as further explanations; the term "risk factor" is not applied to any exposure; comparison drawn with Romanian and international data.	4.1–4.8

Item	Recommendation	How it is addressed	Section
21	Generalisability	Stated that the referral pattern of a county emergency hospital will not generalise to primary-care or screening populations.	4.9
22	Funding	Stated in the back matter.	Funding

Reference: von Elm, E.; Altman, D.G.; Egger, M.; Pocock, S.J.; Gøtzsche, P.C.; Vandenbroucke, J.P. The Strengthening the Reporting of Observational Studies in Epidemiology (STROBE) statement. Lancet 2007, 370, 1453–1457.

Table S2. Items not reportable from the source data

Lesion size, colonic segment, Paris morphology, bowel preparation quality, caecal intubation rate, complications, completeness of resection, TNM stage and treatment, and the behavioural exposures smoking, alcohol and physical activity were not recorded and could not be retrieved. Comorbidity, including diabetes, was likewise not recorded. Each is named in the limitations together with the analysis it prevents. For the 53 patients whose histopathology reports could not be retrieved, no age, sex or month of procedure exists in any available source. The source register holds, beyond the 274 analysed patients, 84 further records with a completed questionnaire but no age, no sex and no histological classification; only 34 of these carry a procedure date, and none can be linked to a particular excluded patient. No comparison between the excluded patients and the analysed cohort is therefore possible. The registry total of procedures performed during the period (n = 978) is the complete unfiltered register and includes examinations of any indication and at any age, examinations that did not reach the caecum, and four procedures later found to have been gastroscopies miscoded as colonoscopies.

Table S3. Lifestyle questionnaire as administered

Self-completed by the patient before the colonoscopy and before any histopathological result was known. Written locally for this study, adapted from no existing instrument, and neither piloted nor validated. Offered by a single gastroenterologist to patients on that physician’s own lists. Translated from Romanian.

Domain	Item	Response options
Anthropometry	Height (cm) and weight (kg)	Free numeric entry; body mass index derived.
Weight change	"In the recent period, have you noticed a significant decrease in weight?"	No / Yes. If yes, kilograms lost in the last 2 weeks, the last month, or the last 3 months. Neither the period nor "significant" was defined for the respondent.
Beverages	Regular coffee consumption	No / Yes; if yes, cups per day (1 / 2–3 / > 3), long or short, taken with milk, taken with sugar.
Beverages	Regular tea consumption	No / Yes; if yes, cups per day (1 / 2–3 / > 3), type (green / black / fruit / herbal), and the type most often used.
Dairy	Regular milk and dairy consumption	No / Yes; if yes, litres per week (1–2 / 2–5 / > 5) and the product most often used.
Meat	Meat consumption	No / Yes; if yes, frequency (once weekly / 3–4 days / daily).
Meat	Processed meat prepared outside the household	No / Yes; if yes, frequency (once weekly / 3–4 days / daily).
Medication	Regular aspirin use	No / Yes; if yes, daily dose (75 mg / 100 mg / other) and duration in years.
Supplements	Regular dietary supplement use	No / Yes; if yes, calcium, zinc or vitamin D with duration, and other supplements as free text.

Domain	Item	Response options
History	Personal history	Adenomatous polyps, inflammatory bowel disease, or cancer, each with year.
History	First-degree family history	Lynch syndrome, familial adenomatous polyposis, juvenile polyposis, Peutz-Jeghers, MYH-associated polyposis; inflammatory bowel disease; cancer.
Presentation	Mode of presentation	Emergency / scheduled.
Presentation	Purpose of presentation	Colorectal cancer screening / symptoms requiring endoscopy / surgical intervention.

Table S4a. Data dictionary for the analysis dataset

Variable	Definition and coding	Recorded (n of 274)
id	Anonymous patient code. Row order randomised; not linkable to any hospital record.	274
age_years	Age in completed years at the time of the procedure.	274
sex	Male / Female.	274
month_of_procedure	Year and month of colonoscopy. Exact dates withheld to prevent re-identification.	274
histology	Most advanced lesion recovered: adenocarcinoma / adenoma / hyperplastic-serrated NFS. NFS = not further specified; serrated lesions were not separately distinguished during the study period. Three lesions carrying the descriptor intramucosal were reclassified as adenoma with high-grade dysplasia before analysis (Section 2.3).	274
architecture	Adenoma or carcinoma architecture: tubular / tubulovillous / villous / NOS. Blank for the three reclassified lesions and for hyperplastic-serrated lesions.	181
grade	Dysplasia grade for adenomas; differentiation grade for carcinomas.	184
tumour_features	Free-text descriptive features of the carcinoma (ulcerated, infiltrative, mucinous differentiation).	38
specimens_submitted	single / multiple. Counts SPECIMENS SUBMITTED TO PATHOLOGY, not lesions seen at endoscopy. Not comparable between histology groups and not used in modelling.	274
synchronous_other_histology	Whether a lesion of a different histological type was recorded in the same patient.	274
advanced_neoplasia	Secondary composite outcome, defined histologically: adenocarcinoma or adenoma with high-grade or focally high-grade dysplasia. Lesion size was unavailable, so the conventional size criterion could not be applied.	274
adenocarcinoma	Primary outcome.	274

Variable	Definition and coding	Recorded (n of 274)
questionnaire_completed	Whether the patient completed the pre-procedure lifestyle questionnaire.	274
height_cm	Self-reported height, centimetres.	63
weight_kg	Self-reported weight, kilograms. Marked digit preference: 57% are multiples of ten.	63
bmi	Body mass index, kg/m², computed from the two fields above.	63
weight_loss	Patient-reported recent weight loss. Item wording: "in the recent period, have you noticed a significant decrease in weight?" Neither the period nor the magnitude was defined for the respondent, and the item did not ask whether the loss was intentional.	78
coffee	Regular coffee consumption (yes/no).	79
tea	Regular tea consumption (yes/no).	78
dairy	Regular milk or dairy consumption (yes/no).	79
meat	Regular meat consumption (yes/no).	79
processed_meat	Regular consumption of processed meat prepared outside the household (yes/no).	79
aspirin	Regular aspirin use (yes/no).	78
supplements	Dietary supplement use (yes/no).	72
family_history_malignancy	Any reported first-degree family history of malignancy (yes/no).	55
coffee_cups_per_day	1 cup / 2-3 cups / > 3 cups.	60
tea_cups_per_day	1 cup / 2-3 cups / > 3 cups.	32
meat_frequency	daily / 3-4 days per week / 1 day per week.	75
processed_meat_frequency	daily / 3-4 days per week / 1 day per week.	50
milk_litres_per_week	1-2 L / 2-5 L / > 5 L.	64
indication_category	Screening / Symptomatic / Other-unspecified, recorded on the questionnaire itself and therefore available only for respondents (75 of 79).	75
coffee_with_milk	Coffee taken with milk (yes/no). Asked only of coffee drinkers.	53
coffee_with_sugar	Coffee taken with sugar (yes/no). Asked only of coffee drinkers.	53
tea_type	Type of tea, from the two options offered on the form: herbal or fruit. Asked only of tea drinkers.	24
tea_type_other	Any other type of tea, written in by the respondent and translated from Romanian. Recorded separately from the item above.	17

Table S4b. De-identified analysis dataset (n = 274) — part 1 of 3 — histopathology

Direct identifiers were removed before analysis. Row order is randomised, exact procedure dates are reduced to year and month, and no record is linkable to any hospital document. Column definitions are in Table S4a. Blank cells denote data not recorded.

id	age_years	sex	month_of_procedure	histology	architecture	grade	tumour_features	specimens_submitted	synchronous_other_histology	advanced_neoplasia	adenocarc[illegible]ma
P001	80	Female	2024-05	adenoma	tubulovillous	high grade dysplasia		single	no	yes	no
P002	67	Male	2023-06	hyperplastic/serrated NFS				multiple	no	no	no
P003	55	Male	2024-03	adenoma	tubulovillous	focally high grade dysplasia		single	yes	yes	no
P004	67	Male	2023-09	adenocarcinoma	tubular	moderately differentiated,		single	no	yes	yes
P005	73	Male	2023-12	adenocarcinoma	NOS	moderately differentiated,	ulcerated	single	no	yes	yes
P006	58	Female	2024-04	adenoma	tubular	low grade dysplasia		multiple	yes	no	no
P007	81	Male	2024-04	hyperplastic/serrated NFS				multiple	no	no	no
P008	57	Female	2024-04	adenoma	tubular	low grade dysplasia		multiple	no	no	no
P009	68	Female	2024-03	adenoma	tubular	focally high grade dysplasia		multiple	yes	yes	no
P010	73	Male	2024-03	adenoma	tubular	low grade dysplasia		single	no	no	no
P011	68	Male	2023-10	adenoma	tubular	low grade dysplasia		multiple	no	no	no
P012	66	Female	2023-11	adenocarcinoma	tubular	moderately differentiated,	infiltrative	single	no	yes	yes
P013	67	Male	2023-12	adenoma	tubular	low grade dysplasia		single	no	no	no
P014	49	Male	2023-11	hyperplastic/serrated NFS				multiple	yes	no	no
P015	62	Female	2024-03	hyperplastic/serrated NFS				single	no	no	no
P016	78	Female	2023-06	adenoma	tubular	high grade dysplasia		multiple	yes	yes	no
P017	79	Male	2023-11	hyperplastic/serrated NFS				multiple	no	no	no
P018	70	Female	2024-03	adenoma	tubular	low grade dysplasia		single	no	no	no
P019	85	Male	2024-04	adenoma	tubulovillous	low grade dysplasia		multiple	no	no	no
P020	62	Female	2023-10	adenoma	tubular	low grade dysplasia		multiple	no	no	no
P021	69	Male	2023-11	adenocarcinoma	tubular	moderately differentiated,	infiltrative	single	no	yes	yes
P022	45	Male	2023-11	adenocarcinoma	tubular	moderately differentiated,	infiltrative	single	no	yes	yes
P023	81	Male	2023-07	adenoma	tubulovillous	low grade dysplasia		multiple	yes	no	no
P024	73	Male	2023-09	hyperplastic/serrated NFS				multiple	no	no	no
P025	71	Male	2023-10	adenoma	tubulovillous	low grade dysplasia		multiple	yes	no	no
P026	68	Male	2024-05	adenoma	tubular	low grade dysplasia		multiple	yes	no	no
P027	77	Male	2023-11	adenocarcinoma	tubular	moderately differentiated,	infiltrative	single	no	yes	yes
P028	55	Male	2024-02	adenoma	tubular	low grade dysplasia		single	no	no	no
P029	50	Male	2024-04	adenoma	tubular	low grade dysplasia		multiple	yes	no	no
P030	67	Female	2023-06	hyperplastic/serrated NFS				single	no	no	no
P031	62	Male	2023-08	adenoma	tubular	high grade dysplasia		multiple	yes	yes	no
P032	46	Female	2023-07	hyperplastic/serrated NFS				single	no	no	no
P033	47	Female	2023-07	hyperplastic/serrated NFS				multiple	no	no	no
P034	56	Male	2023-10	adenocarcinoma	tubular	moderately differentiated,	ulcerated, infiltrative, with focal	single	no	yes	yes
P035	73	Male	2023-08	hyperplastic/serrated NFS				single	no	no	no
P036	54	Male	2024-02	hyperplastic/serrated NFS				multiple	no	no	no
P037	61	Male	2024-03	hyperplastic/serrated NFS				single	no	no	no
P038	62	Female	2024-06	adenoma	tubular	low grade dysplasia		multiple	no	no	no
P039	63	Male	2024-05	adenocarcinoma	NOS	moderately differentiated,	ulcerated	single	no	yes	yes
P040	73	Male	2024-03	adenoma	tubulovillous	focally high grade dysplasia		multiple	yes	yes	no
P041	80	Male	2024-01	adenoma	tubular	low grade dysplasia		multiple	yes	no	no
P042	84	Female	2023-11	adenoma	tubular	low grade dysplasia		multiple	yes	no	no
P043	56	Male	2023-11	adenoma	tubular	low grade dysplasia		multiple	yes	no	no
P044	56	Male	2023-09	adenoma	tubular	low grade dysplasia		multiple	yes	no	no
P045	78	Male	2023-09	hyperplastic/serrated NFS				multiple	no	no	no
P046	47	Male	2024-05	adenoma	tubulovillous	focally high grade dysplasia		single	yes	yes	no
P047	67	Female	2023-10	hyperplastic/serrated NFS				single	no	no	no

id	age_years	sex	month_of_procedure	histology	architecture	grade	tumour_features	specimens_submitted	synchronous_other_histology	advanced_neoplasia	adenocarc[illegible]ma
P048	66	Male	2024-01	adenocarcinoma	NOS	moderately differentiated,	ulcerated	single	yes	yes	yes
P049	57	Female	2024-05	adenoma	tubulovillous	high grade dysplasia		multiple	yes	yes	no
P050	71	Male	2024-05	adenoma	tubulovillous	focally high grade dysplasia		multiple	yes	yes	no
P051	74	Male	2023-12	hyperplastic/serrated NFS				multiple	no	no	no
P052	68	Female	2023-11	hyperplastic/serrated NFS				multiple	no	no	no
P053	54	Male	2024-01	hyperplastic/serrated NFS				multiple	no	no	no
P054	64	Female	2023-08	hyperplastic/serrated NFS				multiple	no	no	no
P055	61	Male	2023-09	adenoma	tubulovillous	low grade dysplasia		single	no	no	no
P056	66	Female	2024-04	adenoma	tubular	low grade dysplasia		single	no	no	no
P057	39	Female	2023-10	adenoma	tubular	low grade dysplasia		multiple	yes	no	no
P058	67	Male	2024-03	adenoma	tubular	low grade dysplasia		multiple	no	no	no
P059	67	Male	2023-10	adenoma	tubulovillous	focally high grade dysplasia		single	yes	yes	no
P060	73	Male	2024-02	hyperplastic/serrated NFS				multiple	no	no	no
P061	47	Female	2023-10	adenoma	tubular	low grade dysplasia		single	no	no	no
P062	78	Female	2024-01	hyperplastic/serrated NFS				multiple	no	no	no
P063	56	Male	2024-05	hyperplastic/serrated NFS				multiple	no	no	no
P064	58	Male	2024-04	hyperplastic/serrated NFS				multiple	no	no	no
P065	67	Female	2023-10	adenoma	tubular	low grade dysplasia		single	no	no	no
P066	78	Male	2023-06	adenoma		high grade dysplasia		multiple	yes	yes	no
P067	66	Male	2024-02	adenocarcinoma	tubular	poorly differentiated, G3	ulcerated	single	no	yes	yes
P068	76	Female	2023-06	adenoma	tubulovillous	low grade dysplasia		single	no	no	no
P069	54	Male	2024-05	hyperplastic/serrated NFS				multiple	no	no	no
P070	56	Female	2024-03	hyperplastic/serrated NFS				single	no	no	no
P071	73	Female	2024-05	hyperplastic/serrated NFS				single	no	no	no
P072	75	Female	2024-05	hyperplastic/serrated NFS				single	no	no	no
P073	75	Male	2024-03	adenoma	tubular	low grade dysplasia		single	no	no	no
P074	74	Male	2024-04	adenoma	tubular	low grade dysplasia		multiple	no	no	no
P075	55	Female	2023-07	adenoma	tubular	low grade dysplasia		single	no	no	no
P076	70	Female	2023-10	hyperplastic/serrated NFS				multiple	no	no	no
P077	52	Male	2024-01	hyperplastic/serrated NFS				single	no	no	no
P078	74	Male	2023-07	hyperplastic/serrated NFS				single	no	no	no
P079	92	Female	2024-03	adenoma	tubulovillous	focally high grade dysplasia		single	yes	yes	no
P080	73	Female	2023-10	hyperplastic/serrated NFS				single	no	no	no
P081	76	Female	2024-02	adenocarcinoma	NOS	moderately differentiated,	ulcerated	single	no	yes	yes
P082	71	Male	2024-05	adenocarcinoma	tubular	moderately differentiated,	infiltrative	single	no	yes	yes
P083	61	Female	2023-07	hyperplastic/serrated NFS				single	no	no	no
P084	76	Male	2023-10	adenoma	tubulovillous	low grade dysplasia		multiple	yes	no	no
P085	83	Male	2023-08	adenocarcinoma	tubular	moderately differentiated,	infiltrative	multiple	yes	yes	yes
P086	78	Male	2024-04	adenocarcinoma	tubular	moderately differentiated,	infiltrative, with focal mucinous	multiple	yes	yes	yes
P087	73	Male	2024-04	adenoma	tubulovillous	focally high grade dysplasia		single	yes	yes	no
P088	55	Male	2023-10	adenocarcinoma	NOS	moderately differentiated,	ulcerated	single	no	yes	yes
P089	50	Female	2023-07	hyperplastic/serrated NFS				single	no	no	no
P090	54	Female	2023-10	hyperplastic/serrated NFS				multiple	no	no	no
P091	65	Male	2024-03	adenoma	tubular	low grade dysplasia		multiple	yes	no	no
P092	67	Male	2024-05	adenoma	tubular	low grade dysplasia		multiple	yes	no	no
P093	69	Male	2023-06	adenoma	tubular	low grade dysplasia		multiple	no	no	no
P094	78	Female	2024-04	adenoma	tubular	low grade dysplasia		multiple	no	no	no
P095	82	Male	2024-03	adenocarcinoma	tubular	moderately differentiated,		multiple	yes	yes	yes
P096	63	Male	2024-01	adenoma	tubular	low grade dysplasia		single	no	no	no
P097	72	Male	2023-11	adenoma	tubulovillous	low grade dysplasia		single	no	no	no
P098	57	Male	2023-08	hyperplastic/serrated NFS				multiple	no	no	no
P099	77	Female	2023-06	hyperplastic/serrated NFS				single	no	no	no
P100	65	Male	2023-11	adenocarcinoma	tubular	moderately differentiated,	infiltrative	single	no	yes	yes
P101	70	Female	2023-10	hyperplastic/serrated NFS				multiple	no	no	no
P102	57	Male	2023-09	adenoma	tubular	low grade dysplasia		multiple	no	no	no

id	age_years	sex	month_of_procedur e	histology	architecture	grade	tumour_features	specimens_subm itted	synchronous_oth er_histology	advanced_neo plasia	adenocarc[illegible] ma
P103	56	Male	2023-12	hyperplastic/serrated NFS				multiple	yes	no	no
P104	60	Male	2023-12	hyperplastic/serrated NFS				single	no	no	no
P105	75	Female	2024-02	hyperplastic/serrated NFS				single	no	no	no
P106	68	Male	2023-06	adenoma	tubulovillous	low grade dysplasia		single	no	no	no
P107	49	Female	2023-07	hyperplastic/serrated NFS				single	no	no	no
P108	63	Female	2023-11	adenoma	tubulovillous	low grade dysplasia		single	no	no	no
P109	80	Male	2023-11	adenoma	tubular	low grade dysplasia		multiple	no	no	no
P110	81	Female	2023-11	adenoma	tubular	low grade dysplasia		multiple	yes	no	no
P111	67	Male	2024-01	adenoma	tubular	low grade dysplasia		multiple	yes	no	no
P112	66	Male	2024-04	adenoma	tubular	low grade dysplasia		multiple	yes	no	no
P113	66	Female	2023-06	adenocarcinoma	NOS	moderately differentiated,	ulcerated	single	no	yes	yes
P114	77	Male	2024-05	adenoma	tubulovillous	focally high grade dysplasia		single	yes	yes	no
P115	77	Male	2024-04	hyperplastic/serrated NFS				multiple	no	no	no
P116	49	Male	2023-08	adenocarcinoma	NOS	moderately differentiated,		single	no	yes	yes
P117	69	Male	2024-04	hyperplastic/serrated NFS				multiple	no	no	no
P118	66	Male	2023-11	adenoma	tubular	low grade dysplasia		multiple	yes	no	no
P119	66	Male	2023-10	adenoma	tubular	low grade dysplasia		single	no	no	no
P120	68	Male	2024-04	adenoma	villous	low grade dysplasia		multiple	yes	no	no
P121	72	Female	2024-03	adenoma	tubular	low grade dysplasia		single	no	no	no
P122	54	Female	2023-10	hyperplastic/serrated NFS				single	no	no	no
P123	54	Female	2023-10	hyperplastic/serrated NFS				multiple	no	no	no
P124	70	Male	2024-03	adenoma	tubulovillous	low grade dysplasia		multiple	yes	no	no
P125	60	Female	2024-06	hyperplastic/serrated NFS				single	no	no	no
P126	73	Female	2024-03	adenocarcinoma	NOS	moderately differentiated,	ulcerated	single	no	yes	yes
P127	56	Male	2023-08	hyperplastic/serrated NFS				multiple	yes	no	no
P128	63	Female	2024-03	adenoma	tubular	low grade dysplasia		single	no	no	no
P129	71	Male	2024-03	adenoma	tubulovillous	focally high grade dysplasia		multiple	yes	yes	no
P130	72	Male	2023-07	adenocarcinoma	tubular	moderately differentiated,		multiple	yes	yes	yes
P131	69	Male	2024-04	adenoma	tubulovillous	low grade dysplasia		multiple	yes	no	no
P132	64	Female	2024-04	hyperplastic/serrated NFS				single	no	no	no
P133	66	Male	2023-06	hyperplastic/serrated NFS				multiple	no	no	no
P134	69	Male	2023-11	adenocarcinoma	tubular	moderately differentiated,	infiltrative	single	no	yes	yes
P135	67	Male	2023-12	adenoma	tubular	low grade dysplasia		single	yes	no	no
P136	68	Male	2023-09	adenocarcinoma	NOS	moderately differentiated,	ulcerated	single	no	yes	yes
P137	69	Female	2024-05	adenocarcinoma	tubular	moderately differentiated,		single	no	yes	yes
P138	67	Male	2023-08	adenoma	tubulovillous	low grade dysplasia		multiple	yes	no	no
P139	57	Male	2023-10	adenoma	tubular	low grade dysplasia		multiple	yes	no	no
P140	70	Female	2023-07	hyperplastic/serrated NFS				single	no	no	no
P141	59	Male	2023-10	adenoma	tubular	low grade dysplasia		multiple	yes	no	no
P142	54	Male	2024-01	adenoma	tubular	low grade dysplasia		single	no	no	no
P143	77	Male	2024-06	adenoma	tubular	low grade dysplasia		multiple	no	no	no
P144	79	Male	2023-10	hyperplastic/serrated NFS				single	no	no	no
P145	79	Female	2023-06	adenocarcinoma	NOS	moderately differentiated,	ulcerated	single	no	yes	yes
P146	63	Male	2024-03	adenocarcinoma	tubular	moderately differentiated,	infiltrative	multiple	yes	yes	yes
P147	64	Female	2024-04	hyperplastic/serrated NFS				multiple	no	no	no
P148	61	Male	2023-12	adenocarcinoma	tubular	moderately differentiated,	infiltrative	single	no	yes	yes
P149	74	Female	2023-09	adenoma	tubular	low grade dysplasia		multiple	yes	no	no
P150	57	Male	2023-06	adenoma	tubular	focally high grade dysplasia		single	yes	yes	no
P151	83	Female	2024-01	hyperplastic/serrated NFS				multiple	no	no	no
P152	73	Male	2023-08	adenoma	tubular	low grade dysplasia		multiple	yes	no	no
P153	75	Male	2023-07	adenoma	tubular	low grade dysplasia		multiple	no	no	no
P154	60	Male	2023-11	adenoma	tubulovillous	low grade dysplasia		single	no	no	no
P155	72	Female	2023-11	hyperplastic/serrated NFS				single	no	no	no
P156	69	Female	2024-01	hyperplastic/serrated NFS				multiple	no	no	no
P157	69	Female	2023-06	adenocarcinoma	tubular	moderately differentiated,	infiltrative	multiple	yes	yes	yes

id	age_years	sex	month_of_procedure	histology	architecture	grade	tumour_features	specimens_submitted	synchronous_other_histology	advanced_neoplasia	adenocar[illegible]ma
P158	64	Male	2024-02	adenoma	tubular	low grade dysplasia		single	no	no	no
P159	68	Female	2023-12	hyperplastic/serrated NFS				single	no	no	no
P160	67	Male	2024-02	hyperplastic/serrated NFS				single	no	no	no
P161	64	Female	2023-11	adenoma	tubular	low grade dysplasia		multiple	no	no	no
P162	66	Male	2024-03	adenoma	tubular	low grade dysplasia		multiple	yes	no	no
P163	60	Female	2023-10	hyperplastic/serrated NFS				single	no	no	no
P164	57	Male	2023-11	adenoma	tubular	low grade dysplasia		multiple	yes	no	no
P165	62	Male	2023-09	hyperplastic/serrated NFS				single	no	no	no
P166	76	Male	2024-01	adenoma	tubular	low grade dysplasia		single	no	no	no
P167	81	Female	2023-11	adenoma	tubular	low grade dysplasia		multiple	no	no	no
P168	67	Female	2023-06	adenoma	tubular	low grade dysplasia		single	no	no	no
P169	66	Male	2024-01	hyperplastic/serrated NFS				multiple	no	no	no
P170	64	Female	2024-02	adenoma	tubular	low grade dysplasia		multiple	yes	no	no
P171	67	Female	2024-05	hyperplastic/serrated NFS				multiple	no	no	no
P172	63	Male	2023-06	adenocarcinoma	tubular	moderately differentiated,	infiltrative	multiple	yes	yes	yes
P173	72	Male	2023-09	hyperplastic/serrated NFS				single	no	no	no
P174	67	Male	2023-08	adenoma	tubular	high grade dysplasia		single	no	yes	no
P175	79	Male	2024-03	adenoma	tubular	focally high grade dysplasia		multiple	yes	yes	no
P176	75	Female	2023-11	adenoma	tubulovillous	focally high grade dysplasia		single	yes	yes	no
P177	69	Male	2024-03	hyperplastic/serrated NFS				single	no	no	no
P178	80	Female	2023-11	hyperplastic/serrated NFS				multiple	no	no	no
P179	72	Female	2024-06	adenoma	tubular	low grade dysplasia		single	no	no	no
P180	56	Female	2023-11	hyperplastic/serrated NFS				multiple	no	no	no
P181	66	Female	2023-09	adenocarcinoma	tubular	moderately differentiated,	infiltrative	single	no	yes	yes
P182	74	Female	2023-07	adenoma	tubular	low grade dysplasia		multiple	yes	no	no
P183	42	Male	2024-05	hyperplastic/serrated NFS				single	no	no	no
P184	63	Male	2024-04	adenoma	tubular	low grade dysplasia		multiple	no	no	no
P185	51	Female	2024-04	adenoma	tubular	low grade dysplasia		single	no	no	no
P186	83	Male	2023-08	adenocarcinoma	NOS	moderately differentiated,	ulcerated	single	no	yes	yes
P187	53	Female	2024-02	adenocarcinoma	NOS	moderately differentiated,	ulcerated	single	no	yes	yes
P188	70	Female	2024-05	adenoma	tubular	low grade dysplasia		multiple	no	no	no
P189	49	Male	2023-06	adenoma	tubular	low grade dysplasia		single	no	no	no
P190	74	Male	2023-11	hyperplastic/serrated NFS				single	no	no	no
P191	75	Female	2023-12	adenoma	tubulovillous	low grade dysplasia		multiple	yes	no	no
P192	56	Female	2023-09	hyperplastic/serrated NFS				multiple	yes	no	no
P193	74	Female	2024-03	adenocarcinoma	NOS	moderately differentiated,	infiltrative	multiple	yes	yes	yes
P194	64	Female	2024-01	adenoma	tubular	focally high grade dysplasia		multiple	yes	yes	no
P195	60	Male	2023-08	adenoma	tubular	low grade dysplasia		multiple	no	no	no
P196	66	Male	2024-03	adenoma	tubulovillous	focally high grade dysplasia		multiple	yes	yes	no
P197	55	Male	2023-08	hyperplastic/serrated NFS				single	no	no	no
P198	88	Male	2023-10	adenoma	tubulovillous	low grade dysplasia		single	no	no	no
P199	72	Male	2023-10	hyperplastic/serrated NFS				single	no	no	no
P200	40	Female	2023-12	hyperplastic/serrated NFS				single	no	no	no
P201	51	Male	2023-08	adenoma	tubular	low grade dysplasia		multiple	no	no	no
P202	73	Male	2024-05	adenoma	tubular	low grade dysplasia		multiple	yes	no	no
P203	73	Female	2023-11	adenoma	tubular	low grade dysplasia		multiple	yes	no	no
P204	84	Male	2023-09	adenoma	tubular	low grade dysplasia		multiple	yes	no	no
P205	71	Female	2023-10	adenoma	tubular	low grade dysplasia		single	no	no	no
P206	60	Female	2023-07	hyperplastic/serrated NFS				multiple	yes	no	no
P207	74	Male	2023-07	adenoma	tubular	low grade dysplasia		multiple	yes	no	no
P208	75	Female	2024-02	adenoma	tubulovillous	focally high grade dysplasia		multiple	yes	yes	no
P209	73	Female	2024-02	adenocarcinoma	tubular	moderately differentiated,	infiltrative	single	no	yes	yes
P210	42	Male	2024-06	adenoma	tubular	low grade dysplasia		multiple	no	no	no
P211	85	Male	2023-06	adenocarcinoma	tubular	moderately differentiated,	infiltrative	single	no	yes	yes
P212	68	Female	2024-05	adenocarcinoma	tubular	moderately differentiated,	infiltrative	single	no	yes	yes

id	age_years	sex	month_of_procedure	histology	architecture	grade	tumour_features	specimens_submitted	synchronous_other_histology	advanced_neoplasia	adenocar[illegible]ma
P213	53	Male	2023-08	adenoma	tubular	low grade dysplasia		multiple	yes	no	no
P214	65	Female	2023-12	adenoma	tubular	low grade dysplasia		single	no	no	no
P215	73	Female	2023-07	adenocarcinoma	tubular	moderately differentiated,		single	no	yes	yes
P216	55	Male	2023-07	adenoma	tubular	low grade dysplasia		multiple	yes	no	no
P217	49	Female	2024-04	adenoma	tubular	focally high grade dysplasia		single	yes	yes	no
P218	72	Male	2024-03	adenoma	tubulovillous	low grade dysplasia		single	no	no	no
P219	69	Male	2023-08	hyperplastic/serrated NFS				single	no	no	no
P220	70	Female	2023-08	adenoma		high grade dysplasia		single	no	yes	no
P221	63	Male	2023-08	hyperplastic/serrated NFS				multiple	no	no	no
P222	74	Male	2024-03	adenoma	tubular	low grade dysplasia		single	no	no	no
P223	71	Female	2023-08	adenocarcinoma	NOS	moderately differentiated,		multiple	yes	yes	yes
P224	78	Male	2023-11	hyperplastic/serrated NFS				single	no	no	no
P225	79	Female	2024-05	adenoma	tubular	low grade dysplasia		multiple	yes	no	no
P226	64	Female	2023-06	adenoma	tubular	low grade dysplasia		multiple	yes	no	no
P227	77	Male	2023-08	adenoma	tubulovillous	low grade dysplasia		multiple	yes	no	no
P228	62	Male	2024-03	adenoma	tubulovillous	low grade dysplasia		multiple	yes	no	no
P229	64	Male	2023-06	adenoma	tubular	low grade dysplasia		single	no	no	no
P230	74	Male	2024-06	adenoma	tubular	low grade dysplasia		multiple	no	no	no
P231	76	Male	2023-11	hyperplastic/serrated NFS				multiple	no	no	no
P232	78	Female	2023-10	hyperplastic/serrated NFS				single	no	no	no
P233	73	Female	2024-03	hyperplastic/serrated NFS				multiple	no	no	no
P234	37	Male	2024-02	hyperplastic/serrated NFS				single	no	no	no
P235	73	Male	2023-10	adenoma	tubular	low grade dysplasia		multiple	yes	no	no
P236	69	Female	2023-07	adenocarcinoma	tubulovillous	moderately differentiated,	infiltrative	single	no	yes	yes
P237	68	Male	2023-06	hyperplastic/serrated NFS				single	no	no	no
P238	43	Male	2024-01	adenocarcinoma	NOS	moderately differentiated,	ulcerated	single	no	yes	yes
P239	62	Male	2024-05	adenoma	tubular	low grade dysplasia		multiple	no	no	no
P240	69	Male	2024-05	adenoma	tubular	low grade dysplasia		multiple	yes	no	no
P241	74	Male	2024-04	adenocarcinoma	NOS	moderately differentiated,	ulcerated	multiple	yes	yes	yes
P242	65	Female	2024-05	adenoma	tubular	low grade dysplasia		multiple	yes	no	no
P243	69	Male	2024-03	adenoma	tubular	low grade dysplasia		multiple	yes	no	no
P244	79	Male	2023-09	adenocarcinoma	tubular	moderately differentiated,	infiltrative	multiple	yes	yes	yes
P245	69	Female	2024-03	hyperplastic/serrated NFS				multiple	no	no	no
P246	75	Male	2023-07	adenoma	tubular	low grade dysplasia		single	no	no	no
P247	83	Male	2023-11	hyperplastic/serrated NFS				single	no	no	no
P248	73	Male	2024-03	adenoma	tubular	low grade dysplasia		multiple	yes	no	no
P249	74	Male	2024-06	adenoma	tubular	low grade dysplasia		multiple	yes	no	no
P250	58	Male	2024-04	adenocarcinoma	tubular	moderately differentiated,	infiltrative	single	yes	yes	yes
P251	73	Female	2024-04	hyperplastic/serrated NFS				single	no	no	no
P252	78	Male	2023-08	adenoma	tubular	low grade dysplasia		multiple	yes	no	no
P253	68	Female	2024-05	adenoma	tubular	low grade dysplasia		single	no	no	no
P254	66	Male	2023-12	adenoma	tubular	low grade dysplasia		single	no	no	no
P255	46	Female	2023-06	hyperplastic/serrated NFS				multiple	no	no	no
P256	54	Male	2023-10	hyperplastic/serrated NFS				single	no	no	no
P257	62	Male	2023-08	adenoma	tubular	low grade dysplasia		multiple	no	no	no
P258	83	Female	2023-11	adenoma		high grade dysplasia		multiple	no	yes	no
P259	77	Female	2024-05	adenocarcinoma	tubular	moderately differentiated,	infiltrative	single	no	yes	yes
P260	61	Female	2023-07	hyperplastic/serrated NFS				single	no	no	no
P261	78	Female	2024-01	adenoma	tubulovillous	focally high grade dysplasia		multiple	no	yes	no
P262	49	Male	2023-08	adenoma	tubulovillous	low grade dysplasia		multiple	yes	no	no
P263	65	Female	2023-11	adenoma	tubular	low grade dysplasia		multiple	no	no	no
P264	74	Male	2024-04	hyperplastic/serrated NFS				multiple	no	no	no
P265	49	Female	2023-11	hyperplastic/serrated NFS				single	no	no	no
P266	71	Male	2023-06	hyperplastic/serrated NFS				single	no	no	no
P267	74	Male	2023-10	adenoma	tubular	low grade dysplasia		multiple	yes	no	no

id	age_years	sex	month_of_procedure	histology	architecture	grade	tumour_features	specimens_submitted	synchronous_other_histology	advanced_neoplasia	adenocarc[illegible] ma
P268	59	Female	2024-04	adenoma	tubulovillous	focally high grade dysplasia		multiple	yes	yes	no
P269	65	Female	2024-03	hyperplastic/serrated NFS				multiple	no	no	no
P270	67	Female	2024-04	hyperplastic/serrated NFS				single	no	no	no
P271	72	Female	2024-04	adenocarcinoma	tubular	moderately differentiated,	infiltrative	single	no	yes	yes
P272	61	Male	2023-06	adenoma	tubular	low grade dysplasia		single	no	no	no
P273	54	Male	2023-07	adenoma	tubular	low grade dysplasia		multiple	yes	no	no
P274	76	Female	2024-05	adenoma	tubular	low grade dysplasia		multiple	yes	no	no

Table S4b. De-identified analysis dataset (n = 274) — part 2 of 3 — questionnaire items

The id column is repeated so the parts can be joined. Blank cells denote items not completed or not applicable.

id	questionnaire_completed	height_cm	weight_kg	bmi	weight_loss	coffee	tea	dairy	meat	processed_meat	aspirin	supplements	family_history_malignancy
P001	no												
P002	yes	184	112	33.1	yes	yes	no	yes	yes	yes	no	no	
P003	no												
P004	yes	170	70	24.2	yes	no	no	yes	yes	yes	no	no	
P005	no												
P006	no												
P007	no												
P008	no												
P009	no												
P010	no												
P011	yes				no	yes	no	yes	yes	yes	no	no	no
P012	yes	160	50	19.5	no	yes	no	yes	yes	yes	no	no	no
P013	no												
P014	yes	167	75	26.9	no	yes	no	no	yes	yes	no	yes	no
P015	no												
P016	yes	160	50	19.5	no	yes	yes	yes	yes	yes	no	no	no
P017	no												
P018	yes	157	80	32.5	no	yes	yes	yes	yes	yes	yes	yes	no
P019	yes				yes	no	no	yes	yes	yes	no	no	no
P020	yes	160	85	33.2	no	yes	no	yes	yes	no	no	yes	yes
P021	no												
P022	no												
P023	yes				no	yes	no	no	yes	yes	no	yes	no
P024	no												
P025	no												
P026	no												
P027	yes				no	yes	yes	no	yes	yes	no	no	no
P028	no												
P029	no												
P030	no												
P031	no												
P032	no												
P033	no												
P034	no												
P035	no												
P036	no												
P037	no												
P038	yes	156	80	32.9	no	yes	yes	yes	yes	yes	no	no	yes
P039	yes	170	60	20.8	yes	no	no	yes	yes	yes	no	no	no
P040	no												
P041	no												
P042	no												
P043	yes	180	73	22.5	no	no	no	yes	yes	no	no	no	no
P044	no												
P045	no												
P046	no												
P047	no												
P048	no												

id	questionnaire_completed	height_cm	weight_kg	bmi	weight_loss	coffee	tea	dairy	meat	processed_meat	aspirin	supplements	family_history_malignancy
P049	no												
P050	no												
P051	no												
P052	yes	173	100	33.4	yes	yes	yes	yes	yes	no	no	no	no
P053	no												
P054	yes	164	90	33.5	no	yes	yes	yes	yes	no	no	no	no
P055	no												
P056	no												
P057	no												
P058	no												
P059	no												
P060	no												
P061	no												
P062	yes	170	70	24.2	no	no	yes	no	yes	no	no	no	no
P063	no												
P064	yes	182	107	32.3	no	yes	no	no	yes	no	no	no	no
P065	no												
P066	no												
P067	no												
P068	yes	159	80	31.6	yes	yes	yes	yes	yes	yes	no	yes	
P069	yes	175	102	33.3	no	yes	no	yes	yes	no	no	no	no
P070	no												
P071	no												
P072	no												
P073	no												
P074	no												
P075	no												
P076	no												
P077	yes	180	88	27.2	no	yes	yes	yes	yes	yes	no	no	yes
P078	yes	172	112	37.9	no	yes	no	no	yes	no	no	no	no
P079	no												
P080	yes	156	82	33.7	no	yes	yes	yes	yes	yes	no	yes	no
P081	no												
P082	no												
P083	yes	152	80	34.6	no	yes	no	yes	yes	yes	yes	no	no
P084	no												
P085	yes	170	70	24.2	yes	no	no	no	yes	yes	no		
P086	no												
P087	no												
P088	no												
P089	no												
P090	no												
P091	no												
P092	yes	185	82	24	no	yes	yes	yes	yes	yes	no	no	
P093	yes	170	80	27.7	no	yes		no	yes	yes	no	no	
P094	no												
P095	yes				no	yes	yes	yes	yes	yes	no	no	no
P096	no												
P097	no												
P098	no												
P099	no												
P100	no												
P101	no												
P102	no												
P103	no												

id	questionnaire_completed	height_cm	weight_kg	bmi	weight_loss	coffee	tea	dairy	meat	processed_meat	aspirin	supplements	family_history_malignancy
P104	no												
P105	yes	159	86	34	no	yes	yes	yes	yes	yes	no	no	yes
P106	no												
P107	no												
P108	no												
P109	no												
P110	yes				no	yes	no	yes	yes	yes	yes	yes	no
P111	no												
P112	no												
P113	yes	160	50	19.5	no	no	yes	no	no	no	no	yes	yes
P114	yes	180	60	18.5	yes	no	yes	yes	yes	yes	no	no	no
P115	no												
P116	no												
P117	no												
P118	no												
P119	no												
P120	no												
P121	yes				no	yes	yes	yes	yes	yes	no	yes	no
P122	no												
P123	yes	156	55	22.6	yes	yes	yes	yes	yes	no	no		
P124	no												
P125	yes	158	76	30.4	no	yes	yes	yes	yes	no	no	no	yes
P126	yes	170	50	17.3	yes	no	no	yes	yes	yes	no	no	no
P127	no												
P128	no												
P129	yes	170	60	20.8	no	yes	yes	yes	yes	no	no	no	no
P130	yes	177	96	30.6	yes	yes	no	yes	yes	yes	yes	no	
P131	no												
P132	no												
P133	yes	170	69	23.9	no	yes	yes	yes	yes	no	no	no	yes
P134	no												
P135	no												
P136	no												
P137	no												
P138	no												
P139	yes	185	120	35.1	no	yes	yes	yes	yes	yes	no	no	yes
P140	no												
P141	no												
P142	no												
P143	no												
P144	no												
P145	yes	165	80	29.4	yes	yes	no	yes	yes	no	no	yes	
P146	yes	170	60	20.8	yes	yes	no	yes	yes	yes	yes	no	no
P147	no												
P148	yes	170	80	27.7	no	yes	yes	yes	no	yes	no	no	no
P149	no												
P150	yes	182	80	24.2	yes	yes	yes	yes	yes	yes	no	no	no
P151	no												
P152	no												
P153	yes	160	55	21.5	yes	yes	no	no	yes	yes	yes		
P154	no												
P155	no												
P156	yes	170	100	34.6	no	yes	no	yes	yes	no	no	no	no
P157	yes	150	80	35.6	yes	yes	yes	yes	yes	no		no	
P158	no												

id	questionnaire_completed	height_cm	weight_kg	bmi	weight_loss	coffee	tea	dairy	meat	processed_meat	aspirin	supplements	family_history_malignancy
P159	no												
P160	no												
P161	no												
P162	no												
P163	no												
P164	no												
P165	yes	180	110	34	no	no	no	yes	yes	no	no	no	no
P166	no												
P167	no												
P168	yes				no	no	no	yes	yes	yes	no	yes	
P169	no												
P170	no												
P171	no												
P172	no												
P173	no												
P174	no												
P175	no												
P176	no												
P177	no												
P178	no												
P179	no												
P180	yes	164	85	31.6	no	yes	no	yes	yes	yes	yes	no	yes
P181	no												
P182	yes	162	84	32	no	yes	no	yes	yes	yes	yes	no	yes
P183	no												
P184	no												
P185	no												
P186	yes				no	no	yes	yes	no	no	no	no	no
P187	yes	160	70	27.3	yes	no	no	yes	yes	yes	no	no	no
P188	yes				no	yes	yes	yes	yes	yes	no	yes	yes
P189	yes	170	130	45	no	yes	no	yes	yes	no	no	no	
P190	no												
P191	no												
P192	yes	155	79	32.9	no	yes	no	no	yes	yes	no	no	no
P193	no												
P194	no												
P195	no												
P196	no												
P197	no												
P198	no												
P199	yes	174	105	34.7	no	yes	yes	yes	yes	yes	no	no	no
P200	no												
P201	no												
P202	no												
P203	no												
P204	no												
P205	no												
P206	no												
P207	no												
P208	no												
P209	no												
P210	yes				no	no	no	yes	yes	yes	no	no	yes
P211	yes	170	80	27.7	yes	no	yes	yes	yes	yes	no		
P212	no												
P213	no												

id	questionnaire_completed	height_cm	weight_kg	bmi	weight_loss	coffee	tea	dairy	meat	processed_meat	aspirin	supplements	family_history_malignancy
P214	no												
P215	yes	152	62	26.8	yes	yes	no	yes	yes	yes	no	no	
P216	yes	165	85	31.2		yes	no	yes	yes	no	yes	yes	yes
P217	yes	160	70	27.3	no	no	no	yes	yes	yes	no	no	no
P218	no												
P219	yes				no	yes	no	yes	yes	no	no		
P220	no												
P221	yes	180	80	24.7	no	yes	no	yes	yes	no	no	no	no
P222	no												
P223	yes	165	70	25.7	no	yes	yes	yes	no	yes	no	no	no
P224	yes	165	78	28.7	no	yes	no	yes	yes	yes	no	no	yes
P225	no												
P226	yes				no	yes	yes	no	yes	no	no		
P227	no												
P228	no												
P229	yes	162	88	33.5	no	yes	no	yes	yes	no	no	no	
P230	no												
P231	no												
P232	no												
P233	no												
P234	no												
P235	no												
P236	no												
P237	no												
P238	no												
P239	no												
P240	no												
P241	no												
P242	no												
P243	no												
P244	yes	170	50	17.3	no	yes	no	yes	yes	no	no		no
P245	no												
P246	yes				no	yes	no	yes	yes	yes	yes	no	yes
P247	no												
P248	no												
P249	no												
P250	yes	180	50	15.4	no	yes	no	no	yes	yes	no	no	no
P251	no												
P252	no												
P253	no												
P254	no												
P255	yes				no	yes	no	yes	yes	yes	no	no	yes
P256	no												
P257	no												
P258	no												
P259	yes	175	90	29.4	no	no	yes	yes	yes	no	no	yes	
P260	yes	160	114	44.5	no	yes	no	yes	yes	no	no	no	
P261	yes	164	100	37.2	yes	yes	no	no	yes	yes	yes	no	
P262	yes	170	98	33.9	yes	no	no	yes	yes	no	no	no	
P263	no												
P264	no												
P265	no												
P266	yes	170	71	24.6	no	yes	no	yes	yes	yes	no	no	
P267	yes				no	yes	yes	yes	yes	yes	no	no	no
P268	no												

id	questionnaire_completed	height_cm	weight_kg	bmi	weight_loss	coffee	tea	dairy	meat	processed_meat	aspirin	supplements	family_history_malignancy
P269	no												
P270	no												
P271	no												
P272	yes	175	90	29.4	no	no	yes	yes	yes	no	no	yes	
P273	no												
P274	no												

Table S4b. De-identified analysis dataset (n = 274) — part 3 of 3 — graded intake, coffee and tea detail, indication

The id column is repeated so the parts can be joined. Blank cells denote items not completed or not applicable.

id	coffee_cups_per_day	coffee_with_milk	coffee_with_sugar	tea_cups_per_day	tea_type	tea_type_other	meat_frequency	processed_meat_frequency	milk_litres_per_week	indication_category
P001										
P002	> 3 cups	no	no				daily	3-4 days per week	2-5 L	Other/unspecified
P003										
P004							3-4 days per week	3-4 days per week	1-2 L	Symptomatic
P005										
P006										
P007										
P008										
P009										
P010										
P011	1 cup	no	no				3-4 days per week	3-4 days per week	1-2 L	Symptomatic
P012	1 cup	no	no				1 day per week	1 day per week	1-2 L	Symptomatic
P013										
P014	2-3 cups	yes	yes				daily	3-4 days per week		Symptomatic
P015										
P016	1 cup	no	no	1 cup	herbal	horsetail/forest fruits	3-4 days per week	daily	1-2 L	Screening
P017										
P018	1 cup	yes	yes	1 cup	herbal		daily	1 day per week	2-5 L	Screening
P019							3-4 days per week	3-4 days per week	1-2 L	Symptomatic
P020	2-3 cups	no	no				3-4 days per week		1-2 L	Symptomatic
P021										
P022										
P023	1 cup	no	no				daily	3-4 days per week		
P024										
P025										
P026										
P027	> 3 cups	yes	no	1 cup	fruit		daily	daily		Symptomatic
P028										
P029										
P030										
P031										
P032										
P033										
P034										
P035										
P036										
P037										
P038	1 cup	yes	no	1 cup	herbal		3-4 days per week	3-4 days per week	1-2 L	Symptomatic
P039							daily	daily	> 5 L	Symptomatic
P040										
P041										
P042										
P043							3-4 days per week		1-2 L	Screening
P044										
P045										
P046										
P047										
P048										

id	coffee_cups_per_day	coffee_with_milk	coffee_with_sugar	tea_cups_per_day	tea_type	tea_type_other	meat_frequency	processed_meat_frequency	milk_litres_per_week	indication_category
P049										
P050										
P051										
P052	1 cup	yes	no	> 3 cups	herbal		3-4 days per week		1-2 L	Screening
P053										
P054	2-3 cups	yes	no	1 cup	herbal	chamomile	1 day per week		2-5 L	Symptomatic
P055										
P056										
P057										
P058										
P059										
P060										
P061										
P062				1 cup	fruit		3-4 days per week			Screening
P063										
P064	2-3 cups	yes	no				daily			Screening
P065										
P066										
P067										
P068	1 cup			2-3 cups	fruit	bilberry/cherry stalks	1 day per week	3-4 days per week	2-5 L	Other/unspecified
P069	2-3 cups	yes	yes				daily		1-2 L	Screening
P070										
P071										
P072										
P073										
P074										
P075										
P076										
P077	1 cup	yes	yes	> 3 cups	herbal	mint/chamomile	3-4 days per week	3-4 days per week	1-2 L	Symptomatic
P078	2-3 cups	no	yes				3-4 days per week			Symptomatic
P079										
P080	1 cup	yes	yes	2-3 cups	herbal	anti-diabetic blend	daily	daily	1-2 L	Symptomatic
P081										
P082										
P083	1 cup	yes	yes				3-4 days per week	3-4 days per week	1-2 L	Symptomatic
P084										
P085							3-4 days per week	3-4 days per week		Other/unspecified
P086										
P087										
P088										
P089										
P090										
P091										
P092	1 cup	no	no	1 cup	fruit		daily	3-4 days per week	1-2 L	Other/unspecified
P093	1 cup	yes	no				3-4 days per week	3-4 days per week		Other/unspecified
P094										
P095	1 cup	no	no	> 3 cups	herbal	sage	daily	daily	1-2 L	Symptomatic
P096										
P097										
P098										
P099										
P100										
P101										
P102										
P103										

id	coffee_cups_per_day	coffee_with_milk	coffee_with_sugar	tea_cups_per_day	tea_type	tea_type_other	meat_frequency	processed_meat_frequency	milk_litres_per_week	indication_category
P104										
P105	1 cup	no	yes	1 cup	herbal	mint	3-4 days per week	3-4 days per week	1-2 L	
P106										
P107										
P108										
P109										
P110	1 cup	no	yes				3-4 days per week	3-4 days per week	1-2 L	Symptomatic
P111										
P112										
P113				> 3 cups		green/black				Screening
P114				2-3 cups			daily	daily	1-2 L	Symptomatic
P115										
P116										
P117										
P118										
P119										
P120										
P121	1 cup	no	yes	1 cup	fruit	herbal	1 day per week	1 day per week	1-2 L	Symptomatic
P122										
P123	2-3 cups	yes	no	1 cup	fruit		daily		1-2 L	Symptomatic
P124										
P125	1 cup	yes	yes	2-3 cups	herbal		3-4 days per week		1-2 L	Symptomatic
P126							daily	daily	1-2 L	Screening
P127										
P128										
P129	1 cup	yes	no	1 cup		nettle	daily		1-2 L	Screening
P130	2-3 cups	no	no				3-4 days per week	3-4 days per week	2-5 L	Other/unspecified
P131										
P132										
P133	2-3 cups	yes	yes	2-3 cups		black	3-4 days per week		1-2 L	Screening
P134										
P135										
P136										
P137										
P138										
P139	1 cup	no	no	1 cup			daily	daily	1-2 L	Symptomatic
P140										
P141										
P142										
P143										
P144										
P145	1 cup	no	no				3-4 days per week		2-5 L	Other/unspecified
P146	1 cup	yes	no				daily	daily	1-2 L	Symptomatic
P147										
P148	1 cup	no	no	1 cup		black		daily	1-2 L	Other/unspecified
P149										
P150	2-3 cups	yes	no	2-3 cups		St John's wort	3-4 days per week	3-4 days per week	1-2 L	Other/unspecified
P151										
P152										
P153	2-3 cups	no	yes				daily	daily		Other/unspecified
P154										
P155										
P156	2-3 cups	no	yes				3-4 days per week		1-2 L	Symptomatic
P157	1 cup	yes	no	2-3 cups	herbal		3-4 days per week			Other/unspecified
P158										

id	coffee_cups_per_day	coffee_with_milk	coffee_with_sugar	tea_cups_per_day	tea_type	tea_type_other	meat_frequency	processed_meat_frequency	milk_litres_per_week	indication_category
P159										
P160										
P161										
P162										
P163										
P164										
P165							daily		1-2 L	Screening
P166										
P167										
P168							3-4 days per week	3-4 days per week	1-2 L	Other/unspecified
P169										
P170										
P171										
P172										
P173										
P174										
P175										
P176										
P177										
P178										
P179										
P180	1 cup	no	yes				3-4 days per week	3-4 days per week	1-2 L	Other/unspecified
P181										
P182	1 cup	yes	yes				3-4 days per week	3-4 days per week	2-5 L	Symptomatic
P183										
P184										
P185										
P186				1 cup		mint			1-2 L	Symptomatic
P187							daily	daily	1-2 L	Symptomatic
P188	2-3 cups	yes	no	1 cup	fruit	herbal	daily	daily	1-2 L	Symptomatic
P189	2-3 cups						daily		1-2 L	Other/unspecified
P190										
P191										
P192	2-3 cups	yes	yes				3-4 days per week	3-4 days per week		Screening
P193										
P194										
P195										
P196										
P197										
P198										
P199	1 cup	yes	yes	2-3 cups	herbal		3-4 days per week	3-4 days per week	1-2 L	Symptomatic
P200										
P201										
P202										
P203										
P204										
P205										
P206										
P207										
P208										
P209										
P210							daily	daily	1-2 L	Symptomatic
P211				1 cup	herbal	mint	3-4 days per week	3-4 days per week	1-2 L	Other/unspecified
P212										
P213										

id	coffee_cups_per_day	coffee_with_milk	coffee_with_sugar	tea_cups_per_day	tea_type	tea_type_other	meat_frequency	processed_meat_frequency	milk_litres_per_week	indication_category
P214										
P215	2-3 cups	yes	no				3-4 days per week	1 day per week	1-2 L	Other/unspecified
P216	1 cup	yes	yes				3-4 days per week		1-2 L	Other/unspecified
P217							daily	3-4 days per week	2-5 L	Symptomatic
P218										
P219	1 cup	no	yes				3-4 days per week		1-2 L	Other/unspecified
P220										
P221	1 cup	no	no				1 day per week		1-2 L	Symptomatic
P222										
P223	> 3 cups							3-4 days per week	1-2 L	Symptomatic
P224	1 cup	no	yes				3-4 days per week	1 day per week	1-2 L	Symptomatic
P225										
P226	2-3 cups			2-3 cups	herbal	St John's wort	daily			Screening
P227										
P228										
P229	2-3 cups						1 day per week		2-5 L	
P230										
P231										
P232										
P233										
P234										
P235										
P236										
P237										
P238										
P239										
P240										
P241										
P242										
P243										
P244	1 cup	no	no				daily		2-5 L	Symptomatic
P245										
P246	1 cup	no	no				3-4 days per week	3-4 days per week	1-2 L	Symptomatic
P247										
P248										
P249										
P250	2-3 cups	no	yes				daily	daily		Symptomatic
P251										
P252										
P253										
P254										
P255	2-3 cups						3-4 days per week	3-4 days per week	1-2 L	Other/unspecified
P256										
P257										
P258										
P259				1 cup	fruit		daily		2-5 L	Other/unspecified
P260	2-3 cups	yes	no				3-4 days per week		1-2 L	Other/unspecified
P261	1 cup						3-4 days per week	1 day per week		Other/unspecified
P262							3-4 days per week		1-2 L	Symptomatic
P263										
P264										
P265										
P266	2-3 cups	no	yes				daily	3-4 days per week	1-2 L	
P267	2-3 cups	no	no	1 cup	herbal		daily	daily	1-2 L	Symptomatic
P268										

id	coffee_cups_per_day	coffee_with_milk	coffee_with_sugar	tea_cups_per_day	tea_type	tea_type_other	meat_frequency	processed_meat_frequency	milk_litres_per_week	indication_category
P269										
P270										
P271										
P272				1 cup	fruit		daily		2-5 L	Other/unspecified
P273										
P274										
